# Supplementary figures and images for: Evolution of a truncated nucleocapsid protein enhances SARS-CoV-2 fitness by suppressing antiviral responses
Source: PLoS Biol. 2026 Apr 1;24(4):e3003646. doi: 10.1371/journal.pbio.3003646 (PMC13043052; doi:10.1371/journal.pbio.3003646)

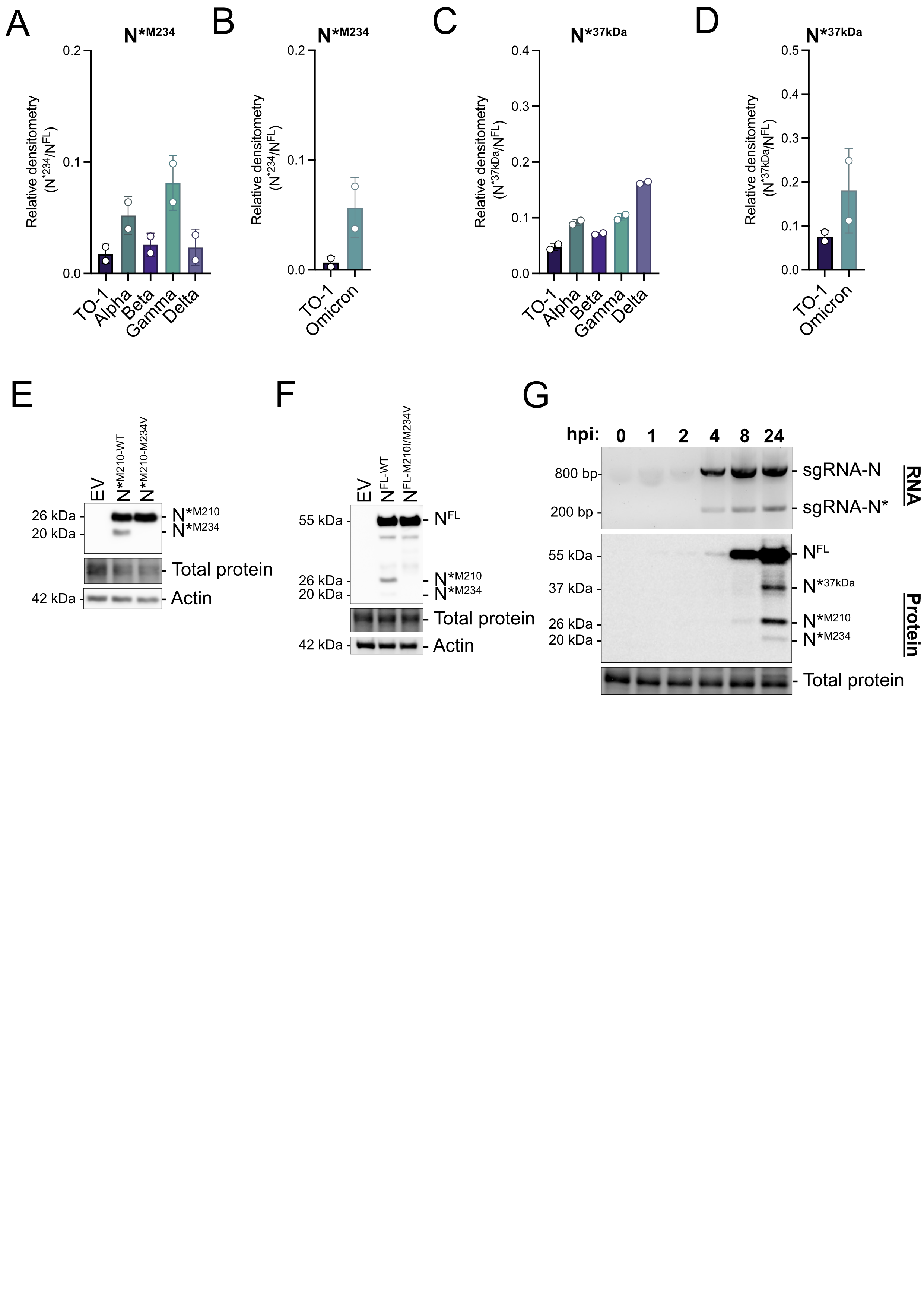

Supplement: S1 Fig — A–D. Protein quantification from Fig 2F (A and B) and H (C and D) was conducted by densitometry. N* proteoform abundance is presented relative to NFL abundance (N*/NFL). These data represent two independent biological replicates (n = 2), SD. See S1 Data for densitometry values. E. N*M210-WT, or N*M210-M234V containing a C-terminal FLAG tag, or an empty vector (EV) control were over-expressed in HEK293T cells and protein lysate was subjected to SDS-PAGE and immunoblotting with anti-FLAG and anti-actin antibodies. See S7 Supporting Data for full blots. F. N*M210-WT, or NFL-M210I/M234V containing a C-terminal FLAG tag, or an empty vector (EV) control were over-expressed in HEK293T cells and protein lysate was subjected to SDS-PAGE and immunoblotting with anti-FLAG and anti-actin antibodies. See S7 Supporting Data for full blots. G. Kinetics of N proteoform and sgRNA profile was determined by infecting Calu3 cells with SARS-CoV-2 Alpha variant (MOI = 4). At the indicated time post-infection, RNA and protein lysate was harvested and was subjected to RT-PCR and agarose gel electrophoresis, and SDS-PAGE and immunoblotting, respectively. See S7 Supporting Data for full blots. (S1_Fig.TIFF) [file pbio.3003646.s001.tiff]

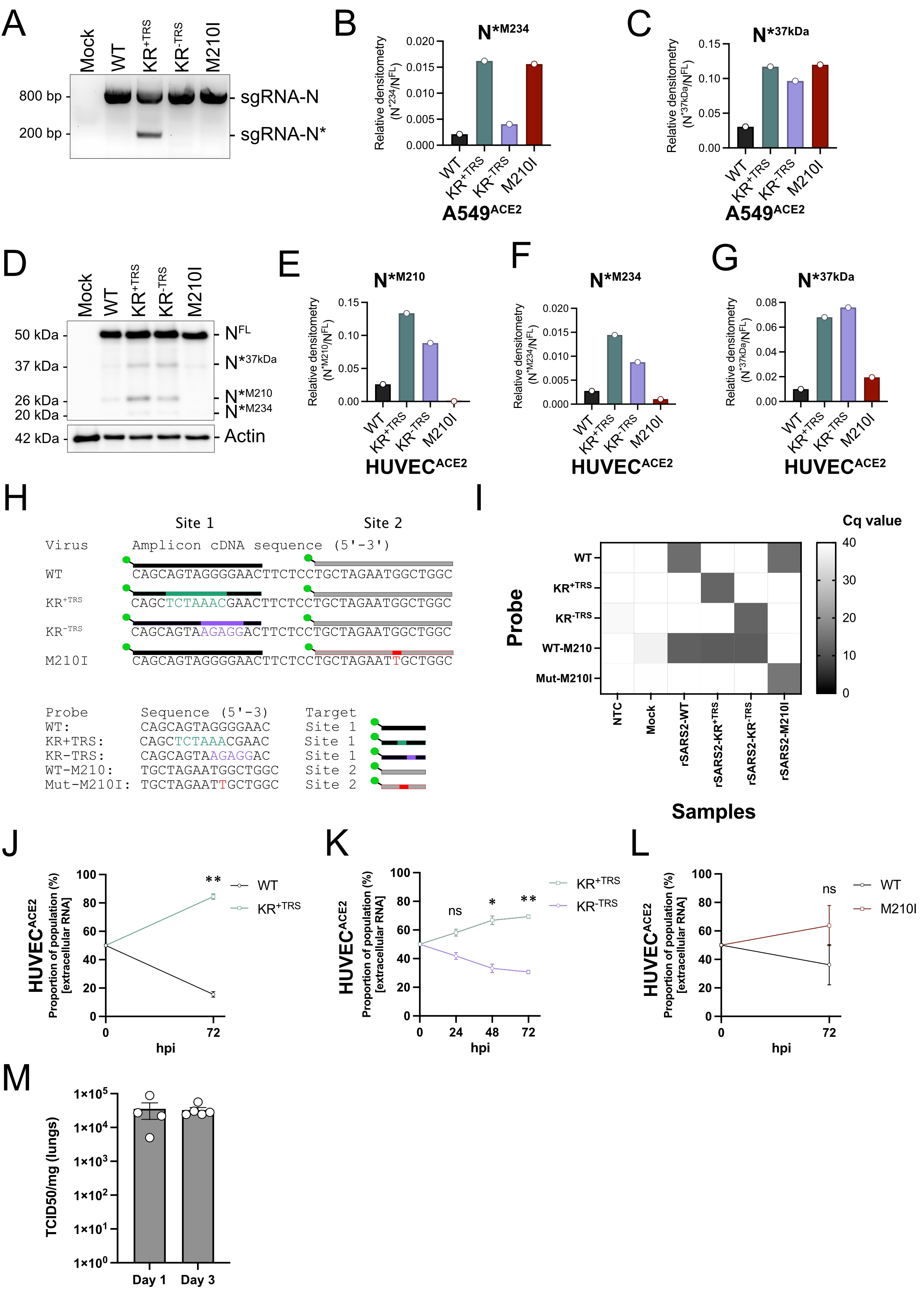

Supplement: S2 Fig — A. sgRNA profile of recombinant SARS-CoV-2 was determined by infecting HUVECACE2 cells (MOI = 4). RNA was harvested 24 hpi and subjected to RT-PCR and agarose gel electrophoresis. See S8 Supporting Data for full gel. B, C. Protein quantification from Fig 3C was conducted by densitometry. N* proteoform abundance is presented relative to NFL abundance (N*/NFL). See S1 Data for densitometry values. D. N proteoform profile of SARS-CoV-2 variants was determined by infecting HUVECACE2 cells (MOI = 4). Protein lysate was harvested 24 hpi and subjected to SDS-PAGE and immunoblotting with anti-N. See S8 Supporting Data for full blots. E–G. Protein quantification from S2D Fig was conducted by densitometry. N* proteoform abundance is presented relative to NFL abundance (N*/NFL). See S1 Data for densitometry values. H. Schematic of DNA probes used to differentiate rSARS-CoV-2 viruses. Probe and respective target sequence to either Site 1 (to differentiate WT, KR+TRS, and KR−TRS) or Site 2 (to differentiate WT from M210I). I. TaqMan probe specificity was validated in RT-qPCR assays using cDNA as a template, generated from RNA from rSARS-CoV-2-infected HUVECACE2 cells (24 hpi, MOI = 4). All possible combinations of probes and recombinant virus-infected samples were tested alongside a no-template control (NTC); resulting Cq values are displayed on a heatmap where reactions without amplification were set to Cq = 40. See S1 Data for quantification values. J–L. Primary HUVECACE2 cells were coinfected with equal infectious titers of the indicated recombinant virus to achieve a total MOI of 0.02. Time 0 represents the inferred proportions of each recombinant based on infectious titer input. At the indicated time post-infection, virus-containing supernatant was harvested, cell debris was removed by centrifugation (5 min at 1,000 RPMs), and extracellular RNA was harvested and subjected to probe-based RT-qPCR to differentiate recombinant virus abundance. These data represent three indepen [file pbio.3003646.s002.tiff]

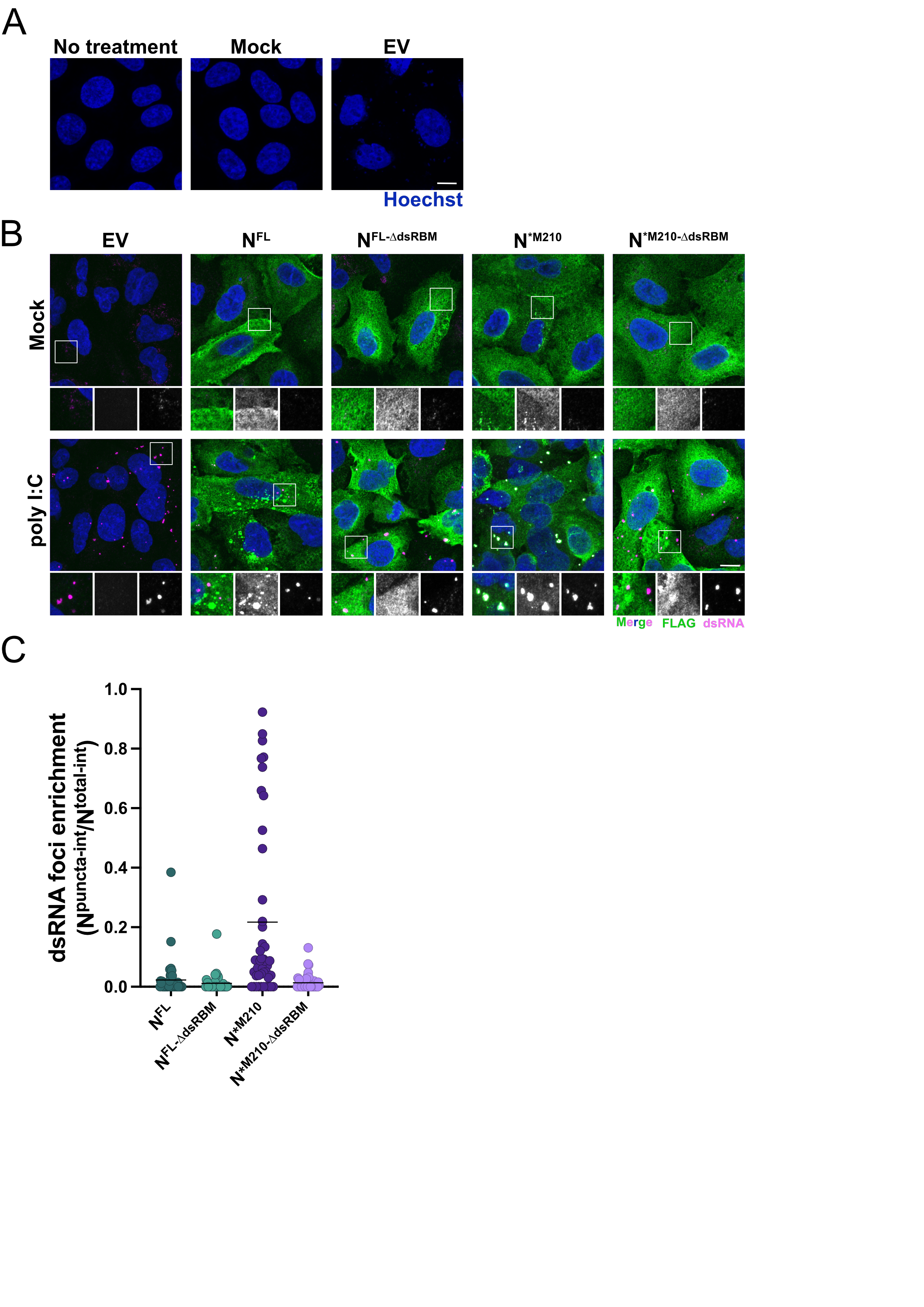

Supplement: S3 Fig — A. HeLa cells were transfected with EV, mock-transfected (treated with Fugene HD only), or not treated. Twenty-four hours post-transfection, cells were fixed, and the nuclei were stained with Hoechst. A maximum intensity projection is presented. Scale bar = 10 µm. B. A549 cells were transduced with recombinant lentiviruses to ectopically express NFL+/-dsRBM or N*M210+/-dsRBM or EV. For all N proteoforms, internal methionine residues were mutated to ensure only the indicated proteoform of N was expressed ([NFL; M210I and M234V], [N*M210; M234V]). Ninety-six hours post-transduction, cells were transfected with 0.5 µg high molecular weight poly I:C or mock-transfected. Three hours post-transfection, cells were fixed and immunostained with the FLAG antibody (N proteoform; Alexa 488) and J2 antibody (dsRNA; Alexa 647). Nuclei were stained with Hoechst. A maximum intensity projection is presented here. One representative experiment of two independent replicates is shown. Scale bar = 10 µm. C. Enrichment of N proteoforms with RNA as in Fig 4D. These data represent two independent biological replicates (n = 2) with 20 cells measured per condition, per replicate. See S1 Data for quantification values. (S3_Fig.TIFF) [file pbio.3003646.s003.tiff]

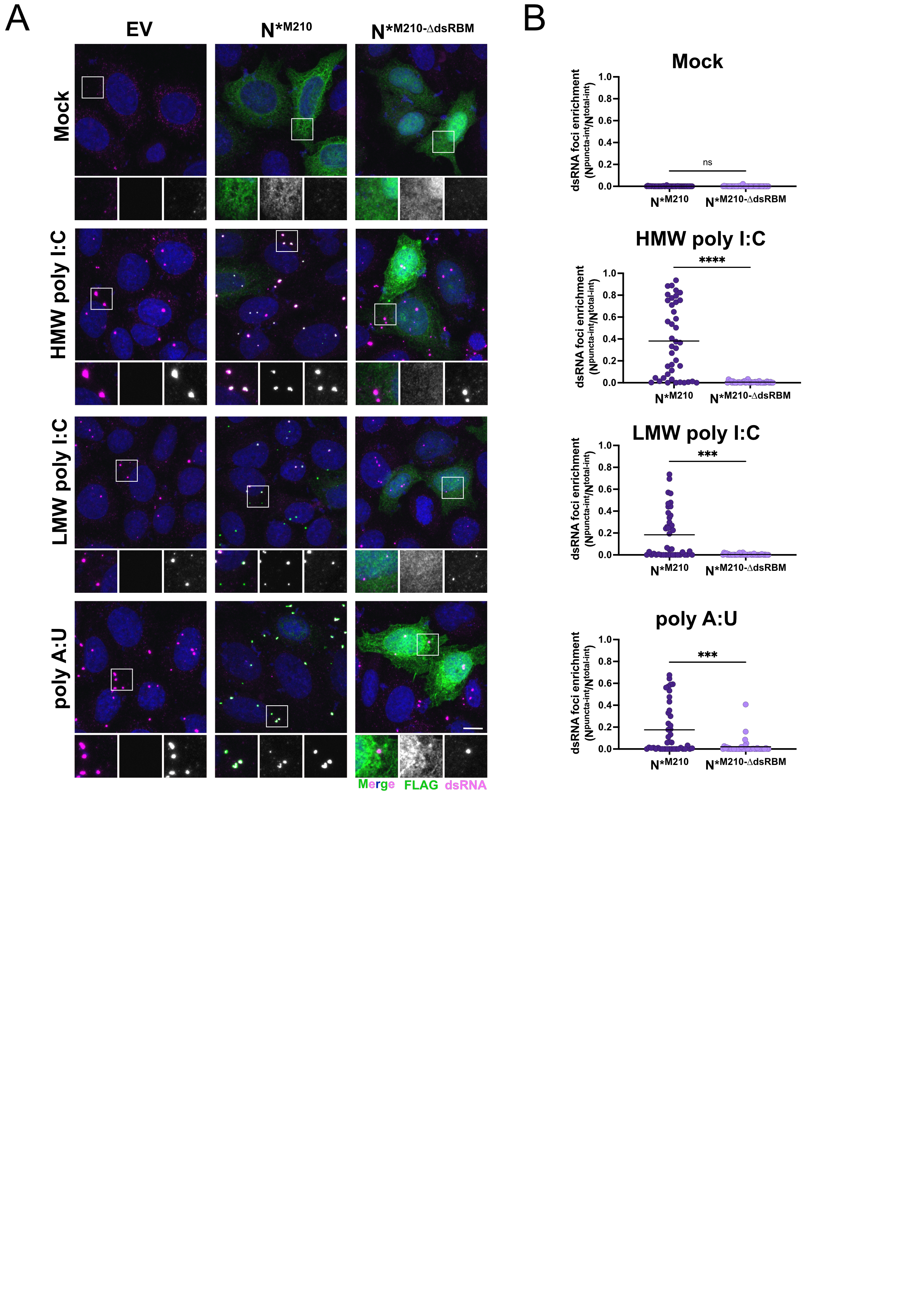

Supplement: S4 Fig — A. EV, N*M210, or N*M210-∆dsRBM-expressing HeLa cells were transfected with 0.5 µg of high molecular weight (HMW), low molecular weight (LMW) poly I:C, or poly A:U, or mock-transfected. Three hours post-transfection, cells were fixed and immunostained with the FLAG antibody (N proteoform; Alexa 488) and J2 antibody (dsRNA; Alexa 647). Nuclei were stained with Hoechst. A maximum intensity projection (MIP) is presented here. One representative experiment of three independent replicates is shown (n = 3). Scale bar = 10 µm. B. Enrichment of N proteoforms with dsRNA as in Fig 4D. These data represent three independent biological replicates (n = 3) with 18 cells measured per condition, per replicate. Statistics were performed using a Mann-Whitney test (*** p < 0.0002, **** p < 0.0001). See S1 Data for quantification values. (S4_Fig.TIFF) [file pbio.3003646.s004.tiff]

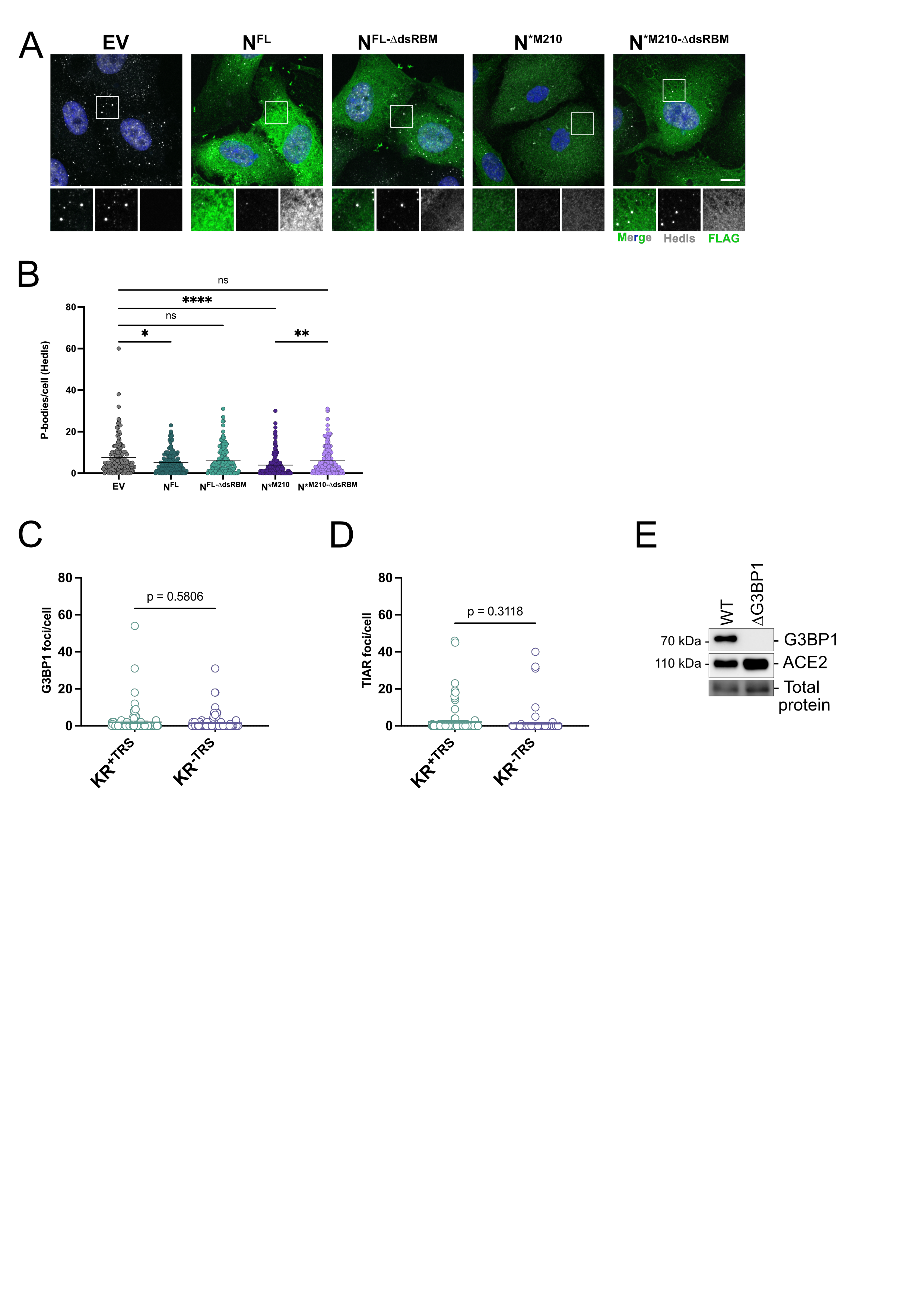

Supplement: S5 Fig — A. Primary HUVEC cells were transduced with recombinant lentiviruses to ectopically express NFL+/-dsRBM, N*M210+/-dsRBM, with internal methionines mutated ([NFL; M210I and M234V], [N*M210; M234V]), or EV. Ninety-six hours post-transduction, cells were fixed and immunostained with the FLAG antibody (N proteoform; Alexa 488) and the Hedls antibody (P-bodies; Alexa 647). Nuclei were stained with Hoechst. A maximum intensity projection (MIP) is presented here. Scale bar = 10 µm. B. P-bodies were quantified using CellProfiler by measuring Hedls puncta in N-expressing cells (thresholded by FLAG staining) or EV transduced cells. These data represent three independent biological replicates (n = 3) with >20 cells measured per condition, per replicate. Each datapoint represents a single cell. Statistics were performed using a Kruskal–Wallis H test with Dunn’s correction (* p < 0.032, ** p < 0.0021, **** p < 0.0001). See S1 Data for quantification values. C, D. Quantification of G3BP1 (C) or TIAR (D) foci from all N-positive cells (combined high and low expressors) from Fig 8A–8E. Foci were quantified using CellProfiler by enumerating G3BP1 or TIAR foci in N-expressing cells. Each datapoint represents a single cell with 100 cells measured per condition. Statistics were performed using an unpaired T test; mean; SEM. See S1 Data for quantification values. E. Protein lysates from wild-type and G3BP1 knockout HEK293AACE2 (∆G3BP1) cells were harvested and resolved by SDS-PAGE and immunoblotted with anti-G3BP1 and anti-ACE2 antibodies. See S9 Supporting Data f or full blots. (S5_Fig.TIFF) [file pbio.3003646.s005.tiff]

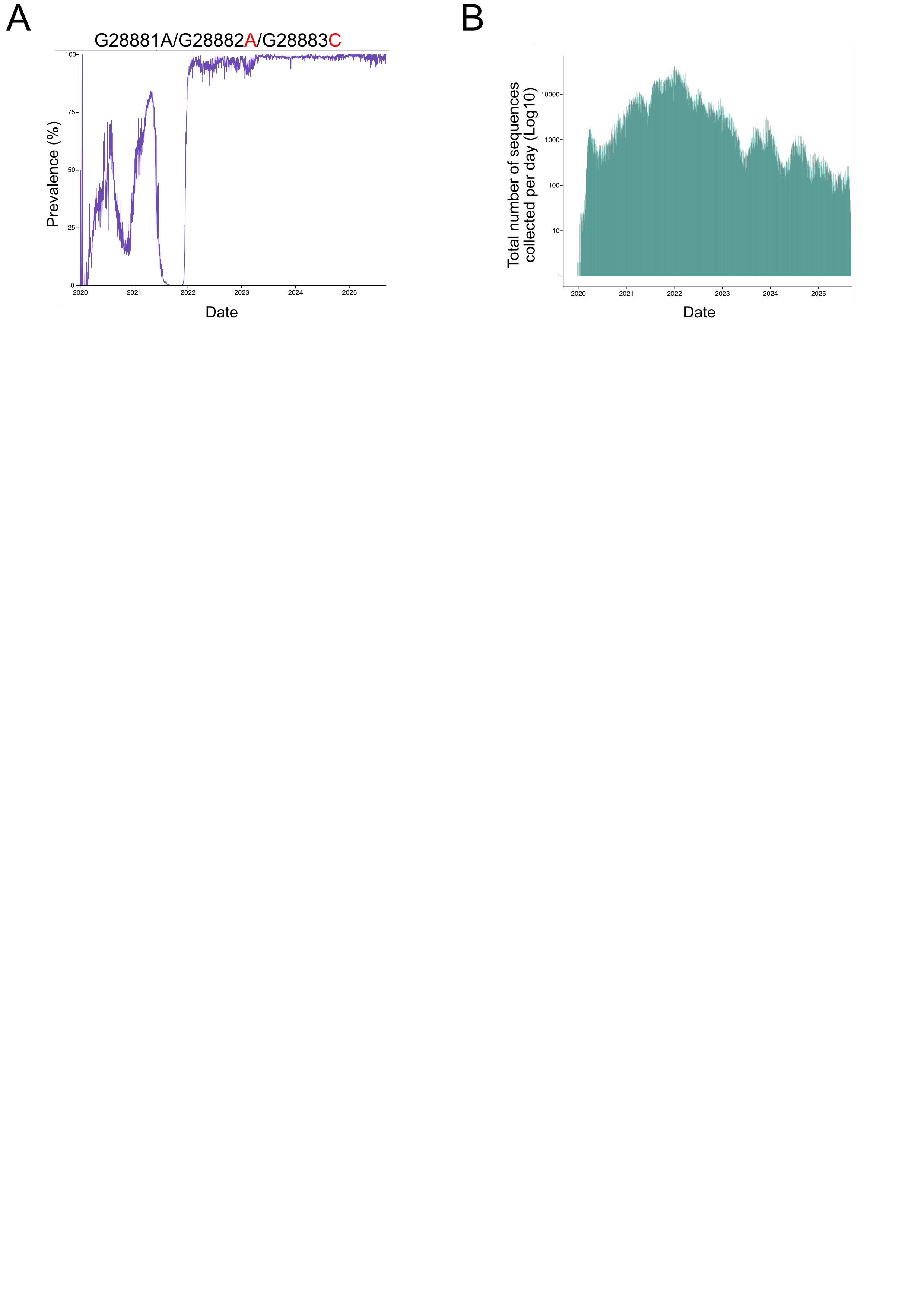

Supplement: S6 Fig — A. The prevalence of the G28881A/G28882A/G28883C mutation was determined as in Fig 9C and the genomes with the G28881A/G28882A/G28883C mutation were plotted over time. Here, sequences collected from all days are plotted, not just days with >30 sequences collected per day as in Fig 9C. 28882A and 28883C are highlighted in red as they contributed to the N* TRS. B. The number of sequences collected per day were extracted from Nextstrain.org showing the low sequencing coverage at the end of 2019 and start of 2020. (S6_Fig.TIFF) [file pbio.3003646.s006.tiff]

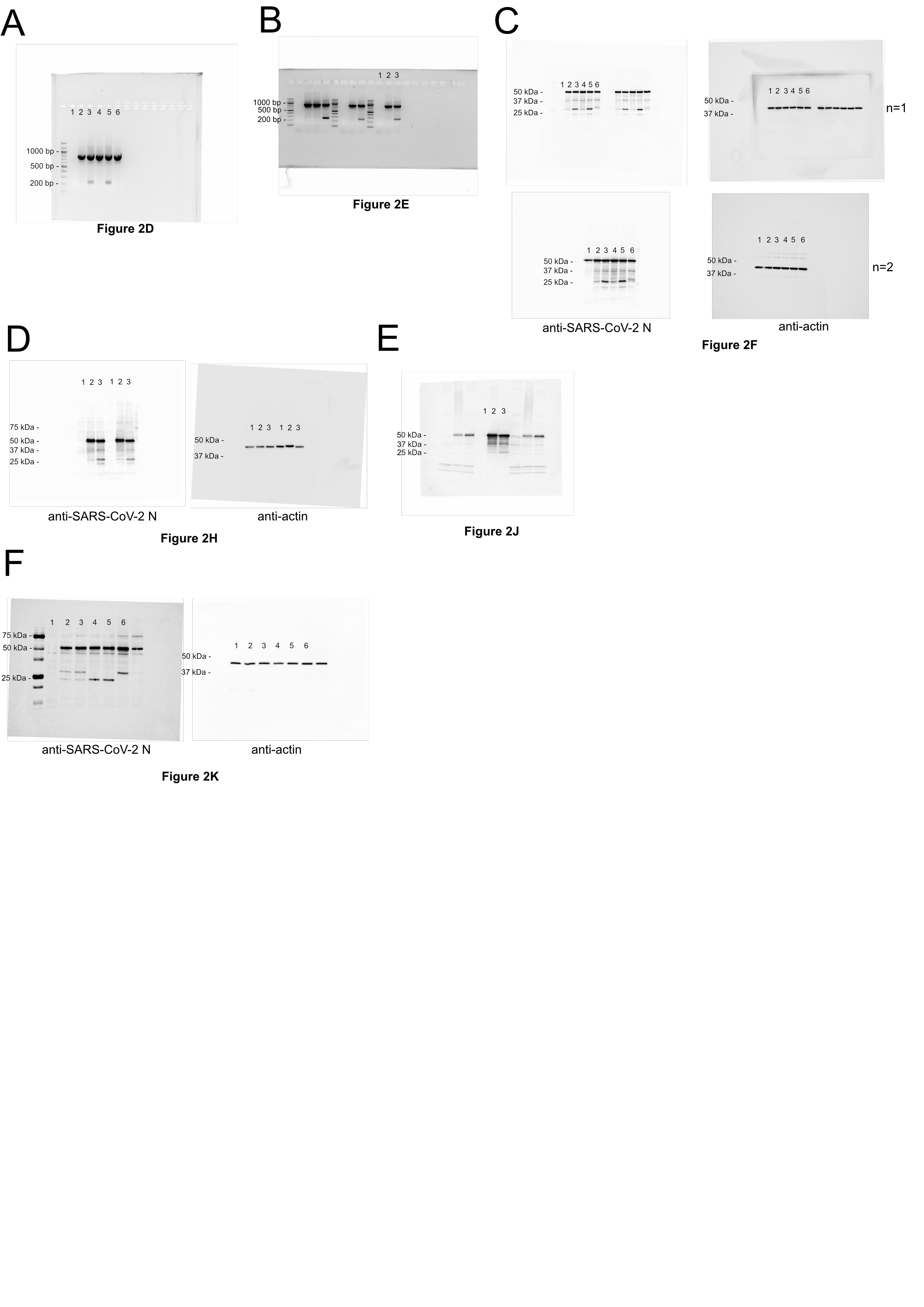

Supplement: S1 Supporting Data — A and B. Agarose gel showing sgRNA-N* synthesis from SARS-CoV-2 variant infections. A. Lane 1 = mock-infected, Lane 2 = TO-1, Lane 3 = Alpha, Lane 4 = Beta, Lane 5 = Gamma, Lane 6 = Delta. B. Lane 1 = mock-infected, Lane 2 = TO-1, Lane 3 = Omicron BA1. C–E. Immunoblot showing the synthesis of N* proteoforms from SARS-CoV-2 variant infections. C. Lane 1 = mock-infected, Lane 2 = TO-1, Lane 3 = Alpha, Lane 4 = Beta, Lane 5 = Gamma, Lane 6 = Delta. D. Lane 1 = mock-infected, Lane 2 = TO-1, Lane 3 = Omicron BA1. E. And the presence of N* in isolated virus particles; Lane 1 = mock-infected, Lane 2 = TO-1, Lane 3 = Alpha. F. NFL over-expression with various methionine mutations. Lane 1 = EV, Lane 2 = NFL-WT, Lane 3 = NFL-M101G, Lane 4 = NFL-M210T, Lane 5 = NFL-M210I, Lane 6 = NFL-M234V. (S1_Supporting Data.TIFF) [file pbio.3003646.s008.tiff]

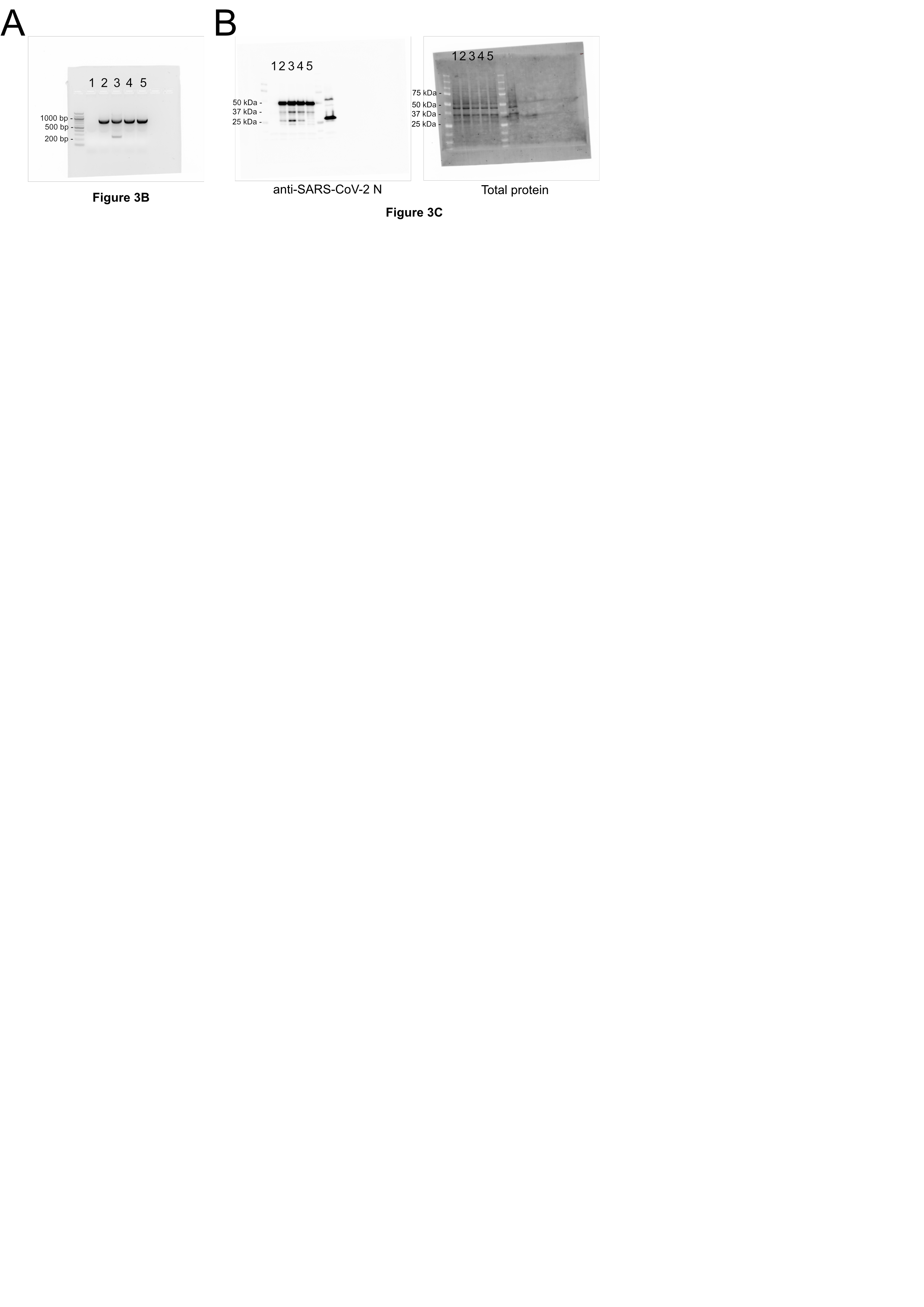

Supplement: S2 Supporting Data — A. Agarose gel showing sgRNA-N* synthesis from recombinant SARS-CoV-2 following infection of A549ACE2 cells. A. Lane 1 = mock-infected, Lane 2 = WT, Lane 3 = KR+TRS, Lane 4 = KR−TRS, Lane 5 = M210I. B. Immunoblot showing the synthesis of N* proteoforms from recombinant SARS-CoV-2 following infection of A549ACE2 cells. Lane 1 = mock-infected, Lane 2 = WT, Lane 3 = KR+TRS, Lane 4 = KR−TRS, Lane 5 = M210I. (S2_Supporting Data.TIFF) [file pbio.3003646.s009.tiff]

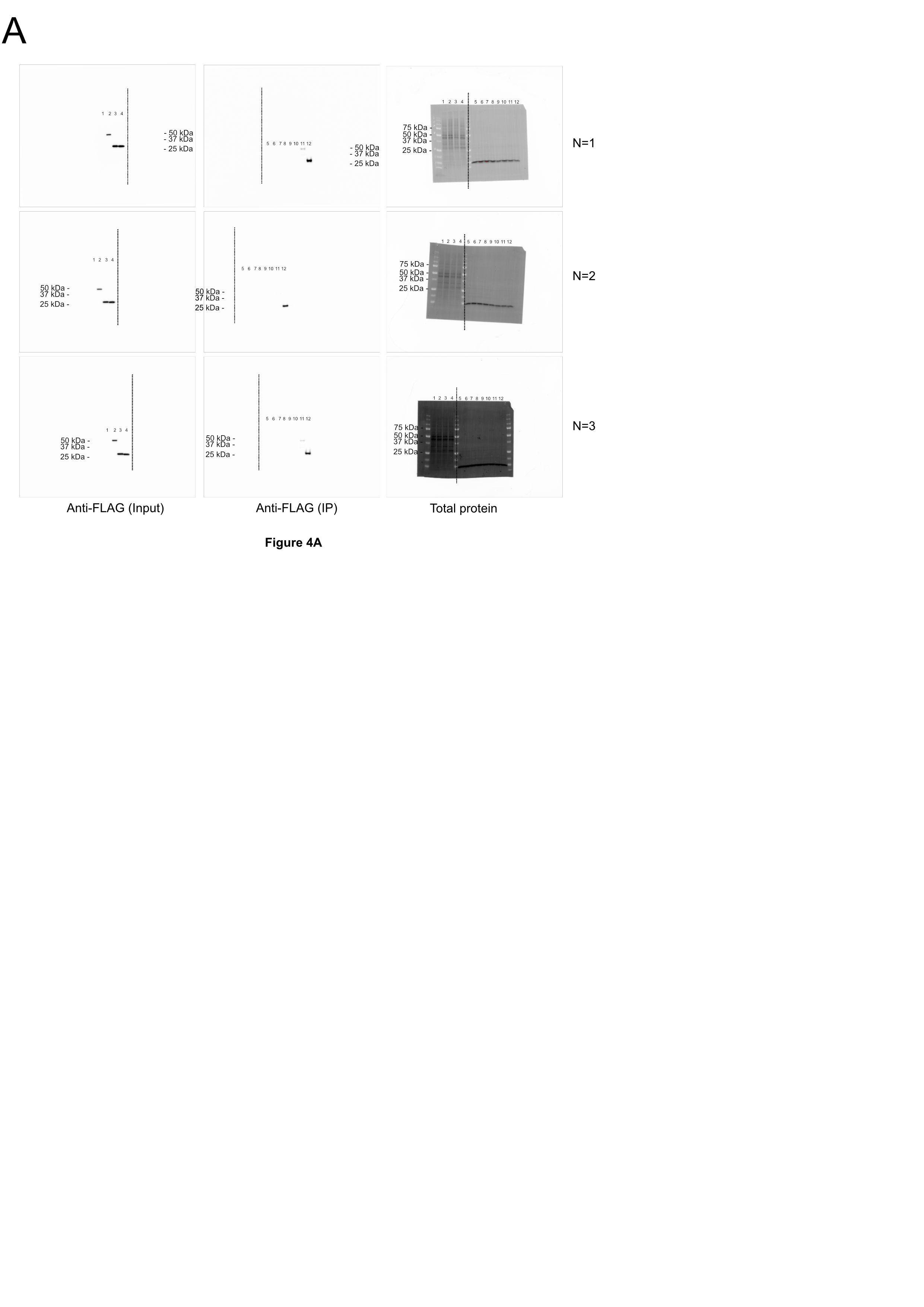

Supplement: S3 Supporting Data — A. Immunoblots from poly I:C immuno-precipitation. Lanes 1–4 = input (Lane 1 = EV, Lane 2 = NFL, Lane 3 = N*M210, Lane 4 = N*M210-ΔdsRBM); Lanes 5–8 = pulldown with no poly I:C (Lane 5 = EV, Lane 6 = NFL, Lane 7 = N*M210, Lane 8 = N*M210-ΔdsRBM); Lanes 9–12 = pulldown with poly I:C (Lane 9 = EV, Lane 10 = NFL, Lane 11 = N*M210, Lane 12 = N*M210-ΔdsRBM). (S3_Supporting Data.TIFF) [file pbio.3003646.s010.tiff]

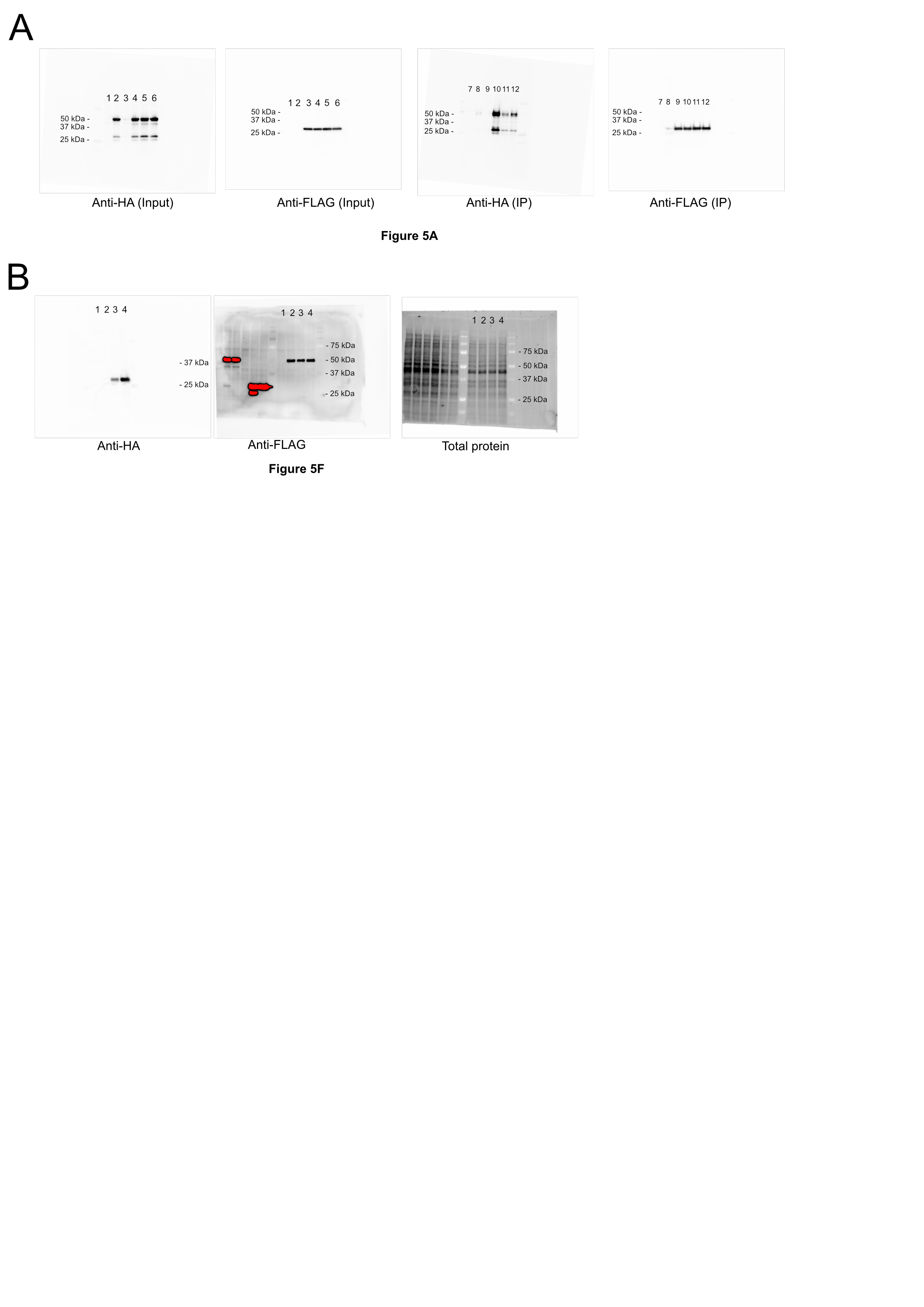

Supplement: S4 Supporting Data — A. Immunoblot from NFL-WT-HA:N*M210-FLAG co-IP. Lanes 1–6 = input (Lane 1 = EV, Lane 2 = NFL-WT-HA, Lane 3 = N*M210-FLAG, Lane 4 = NFL-WT-HA:N*M210-HA, Lane 5 = NFL-WT-HA:N*M210-HA with 10 μg/mL RNase A, Lane 6 = NFL-WT-HA:N*M210-HA with 50 μg/mL RNase A); Lanes 7–12 = IP (Lane 7 = EV, Lane 8 = NFL-WT-HA, Lane 9 = N*M210-FLAG, Lane 10 = NFL-WT-HA:N*M210-HA, Lane 11 = NFL-WT-HA:N*M210-HA with 10 μg/mL RNase A, Lane 12 = NFL-WT-HA:N*M210-HA with 50 μg/mL RNase A). B. Co-expression of NFL-FLAG and N*M210-HA. Lane 1 = EV, Lane 2 = NFL-FLAG, Lane 3 = NFL-FLAG:N*M210-HA [8:1], Lane 4 = NFL-FLAG:N*M210-HA [2:1]. (S4_Supporting Data.TIFF) [file pbio.3003646.s011.tiff]

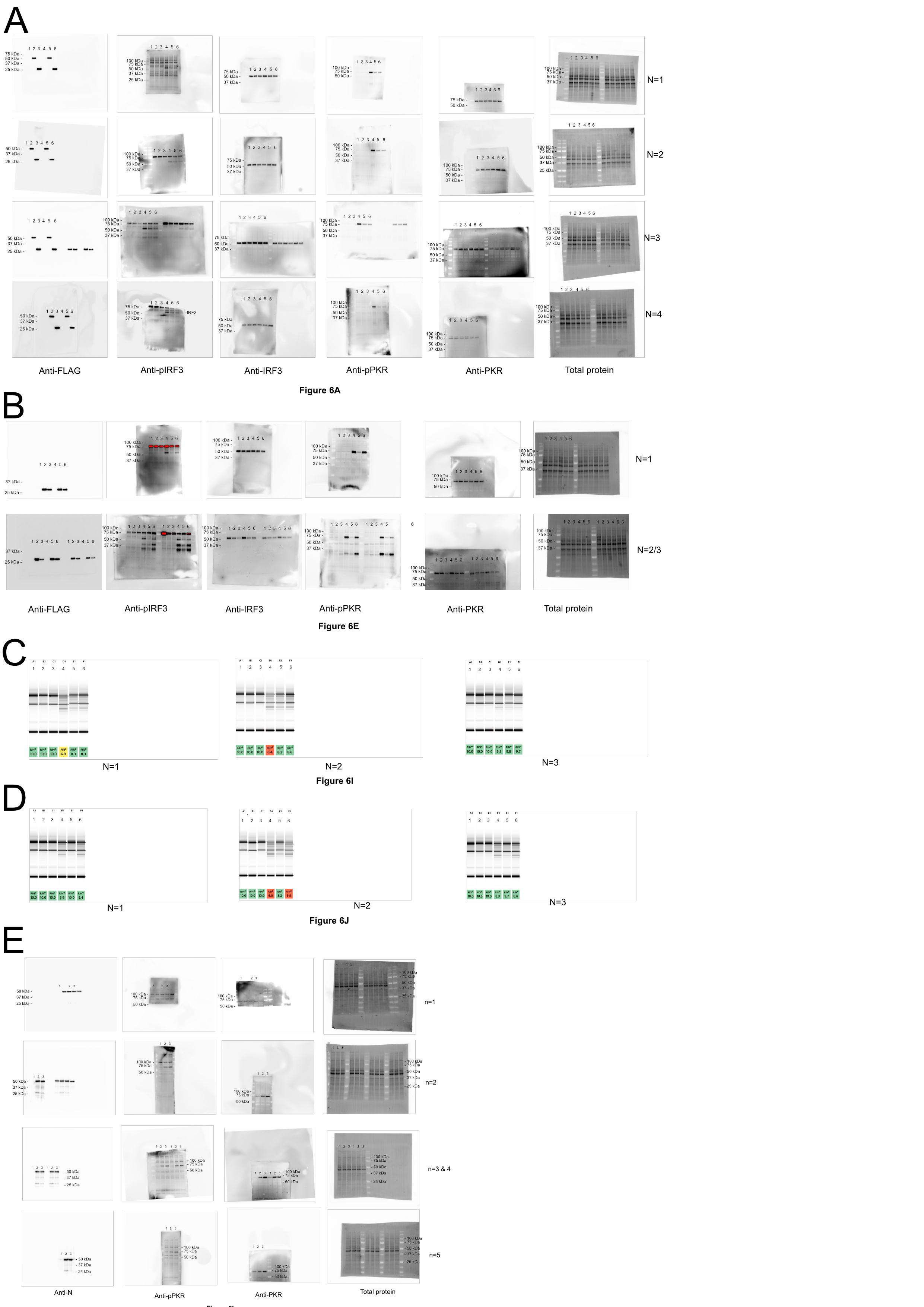

Supplement: S5 Supporting Data — A. Immunoblots showing NFL/N*M210 inhibition of poly I:C-induced immune responses. Lanes 1–3 = mock-treated (Lane 1 = EV, Lane 2 = NFL, Lane 3 = N*M210); Lanes 4–6 = poly I:C-treated (Lane 4 = EV, Lane 5 = NFL, Lane 6 = N*M210). B. Immunoblots showing N*M210 inhibition of poly I:C-induced immune responses. Lanes 1–3 = mock-treated (Lane 1 = EV, Lane 2 = N*M210, Lane 3 = N*M210-ΔdsRBM); Lanes 4–6 = poly I:C-treated (Lane 4 = EV, Lane 5 = N*M210, Lane 6 = N*M210-ΔdsRBM). C. TapeStation rRNA integrity automated electrophoresis. Lanes 1–3 = mock-treated (Lane 1 = EV, Lane 2 = NFL, Lane 3 = N*M210); Lanes 4–6 = poly I:C-treated (Lane 4 = EV, Lane 5 = NFL, Lane 6 = N*M210). D. TapeStation rRNA integrity automated electrophoresis. Lanes 1–3 = mock-treated (Lane 1 = EV, Lane 2 = N*M210, Lane 3 = N*M210-ΔdsRBM); Lanes 4–6 = poly I:C-treated (Lane 4 = EV, Lane 5 = N*M210, Lane 6 = N*M210-ΔdsRBM). E. Immunoblot showing KR+TRS and KR−TRS immune activation. Lane 1 = mock-infected, Lane 2 = KR+TRS, Lane 3 = KR−TRS. (S5_Supporting Data.TIFF) [file pbio.3003646.s012.tiff]

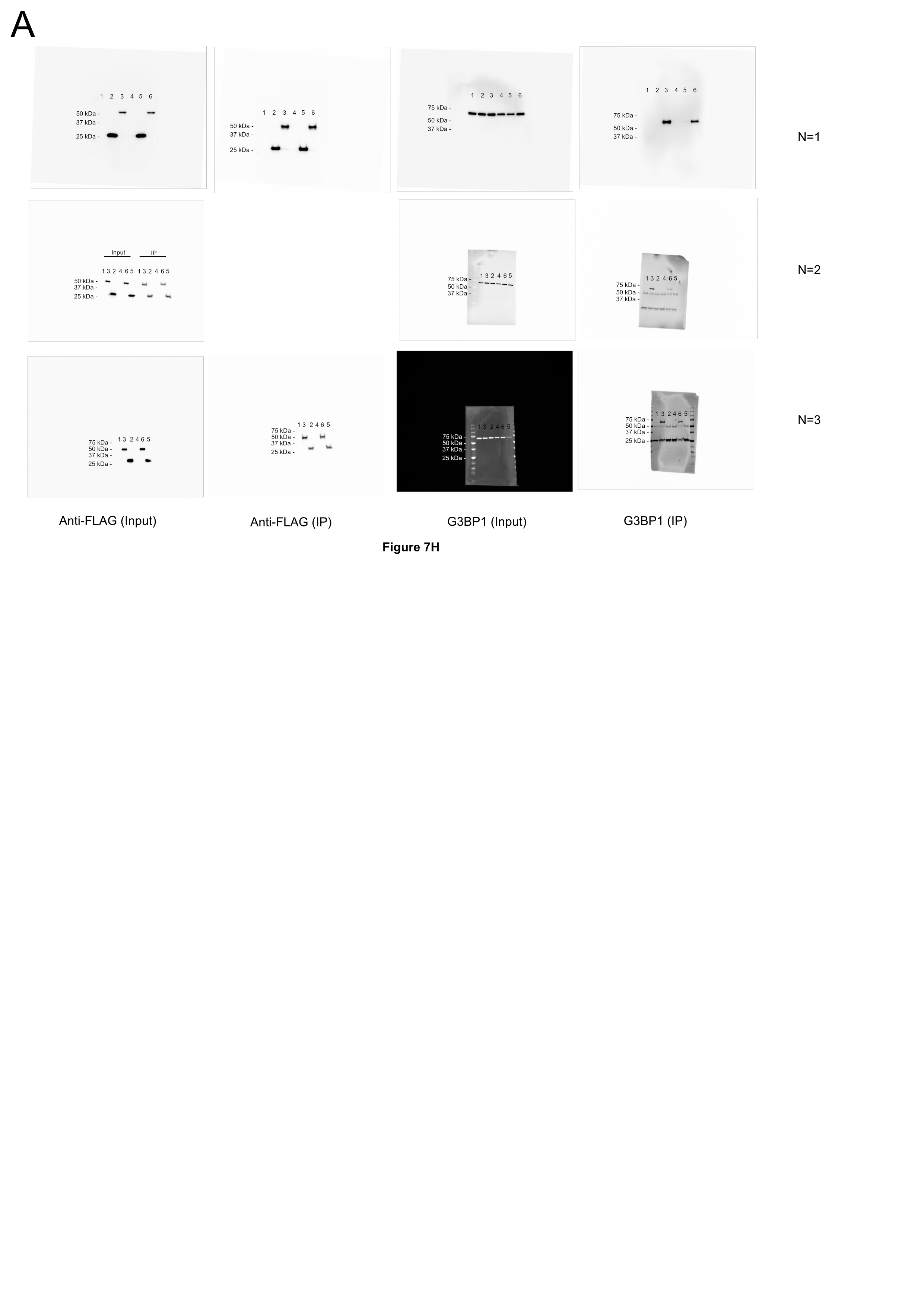

Supplement: S6 Supporting Data — A. Immunoblot showing NFL-G3BP1 co-immunoprecipitation. Lanes 1–3 = mock-treated (Lane 1 = EV, Lane 2 = N*M210, Lane 3 = NFL); Lanes 4–6 = poly I:C-treated (Lane 4 = EV, Lane 5 = N*M210, Lane 6 = NFL). (S6_Supporting Data.TIFF) [file pbio.3003646.s013.tiff]

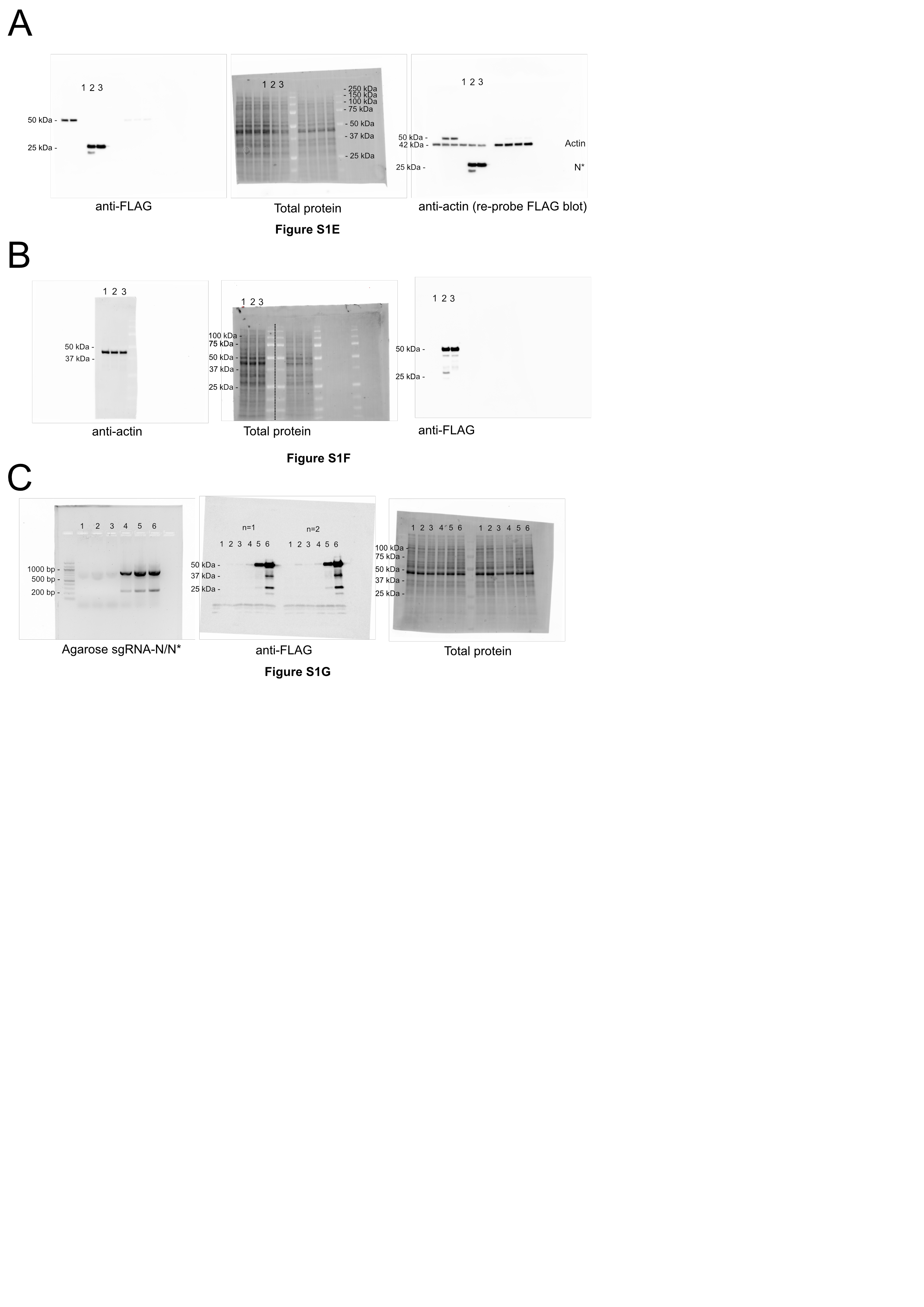

Supplement: S7 Supporting Data — A. Lane 1 = EV, Lane 2 = N*M210-WT, Lane 3 = N*M210-M234V. B. Lane 1 = EV, Lane 2 = NFL-WT, Lane 3 = NFL-M210I/M234V. C. Agarose gel and immunoblot of sgRNA-N* and N*M210 synthesis kinetics. Lane 1 = 0 hpi, Lane 2 = 1 hpi, Lane 3 = 2 hpi, Lane 4 = 4 hpi, Lane 5 = 8 hpi, Lane 6 = 24 hpi. (S7_Supporting Data.TIFF) [file pbio.3003646.s014.tiff]

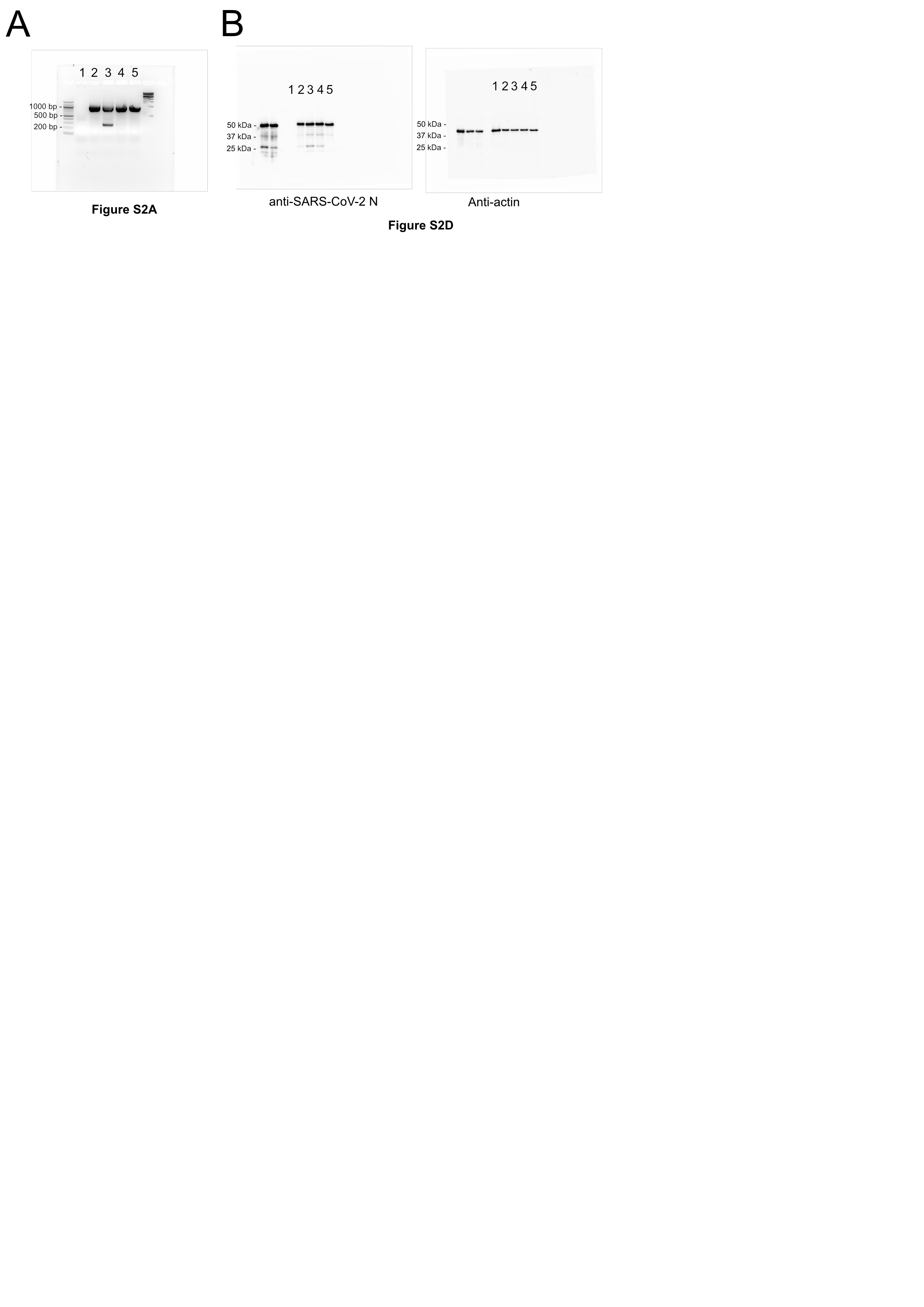

Supplement: S8 Supporting Data — A. Agarose gel showing sgRNA-N* synthesis from recombinant SARS-CoV-2 following infection of HUVECACE2 cells. A. Lane 1 = mock-infected, Lane 2 = WT, Lane 3 = KR+TRS, Lane 4 = KR−TRS, Lane 5 = M210I. B. Immunoblot showing the synthesis of N* proteoforms from recombinant SARS-CoV-2 following infection of HUVECACE2 cells. Lane 1 = mock-infected, Lane 2 = WT, Lane 3 = KR+TRS, Lane 4 = KR−TRS, Lane 5 = M210I. (S8_Supporting Data.TIFF) [file pbio.3003646.s015.tiff]

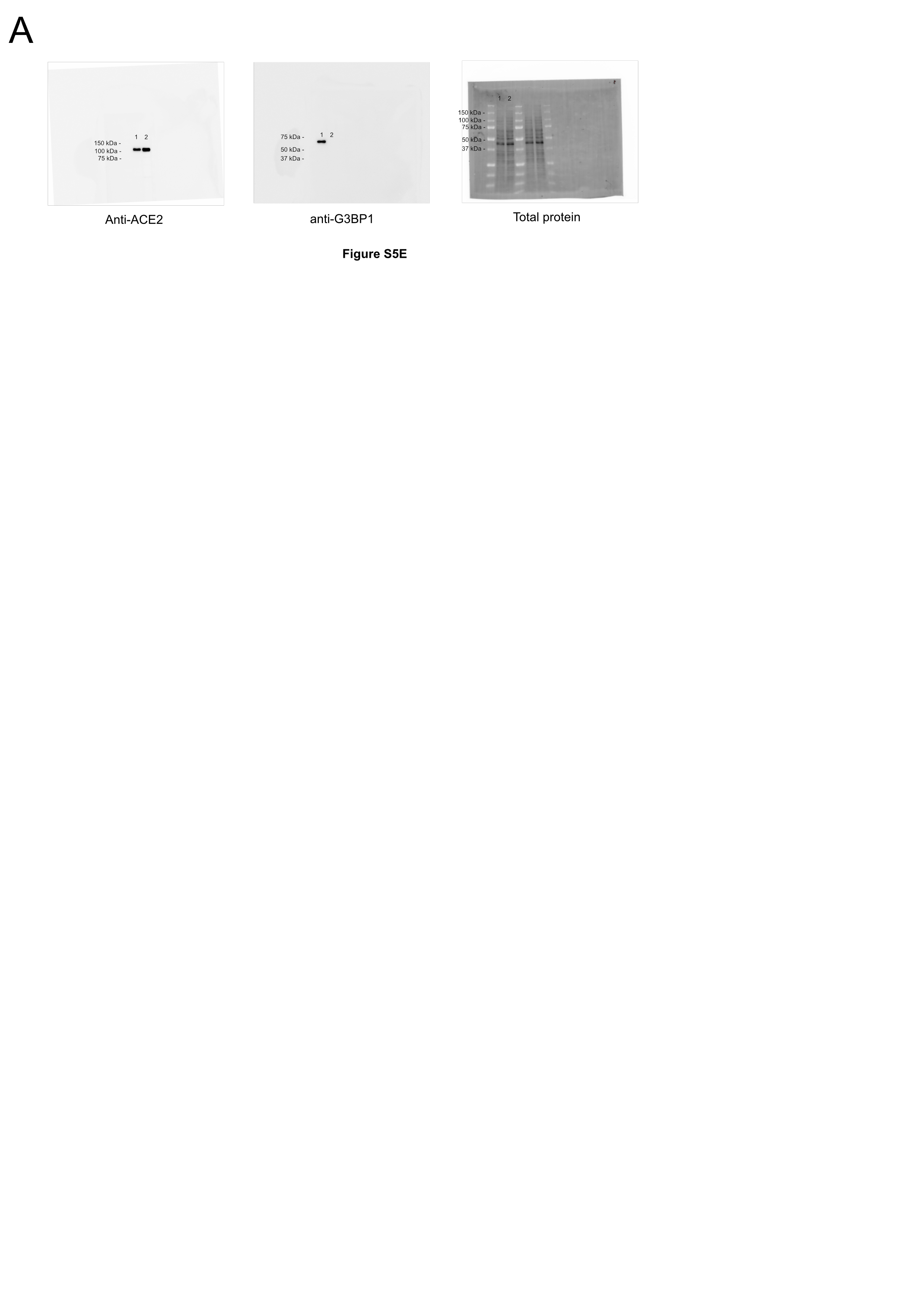

Supplement: S9 Supporting Data — A. Immunoblot validation of G3BP1 expression. Lane 1 = wild-type HEK293AACE2, Lane 2 = HEK293AACE2-ΔG3BP1. (S9_Supporting Data.TIFF) [file pbio.3003646.s016.tiff]
