# Supplementary material for: Evolution of a truncated nucleocapsid protein enhances SARS-CoV-2 fitness by suppressing antiviral responses
Source: PLoS Biol. 2026 Apr 1;24(4):e3003646. doi: 10.1371/journal.pbio.3003646 (PMC13043052; doi:10.1371/journal.pbio.3003646)
Supplement: Attachment — Submitted filename: Mulloy et al Response to reviewers Final.pdf [file pbio.3003646.s019.pdf]

<u>Response to Reviewers</u>

<u>Reviewer 1 Comments</u>

This manuscript examines the function of a SARS-2 NP variant synthesized from a novel sgRNA species. The main conclusion is that SARS-2 variants synthesize a subgenomic RNA encoding a C-term NP truncation, and this feature specifically enhances viral fitness during experiments with separation of function recombinant viruses. A second conclusion is that this NP variant binds dsRNA and inhibits dsRNA immune responses, dependent upon the ability to bind dsRNA. These observations are well supported and are worthy of publication.
*The third conclusion is that N*M210 inhibits stress granule formation and that this activity is the basis of improved viral fitness. While this may be true, one would need additional experiments to be convinced of this mechanistic interpretation, as detailed below. This conclusion should be modified.*

We thank Dr. Parker for his appreciation of our data and for this insightful and thorough review. We have addressed Dr. Parker's comments about the third conclusion by adding new experimental data and by tempering the strength of our statements. In response to these changes, we believe the manuscript is now significantly improved.

The biggest issue with this manuscript is the interpretation that the fitness advantage of the M120 protein comes, at least in part, from inhibiting stress granules. As detailed below, I have several issues with this conclusion. My suggestion is that authors note the impacts of Nfl and NM120 on stress granules and P-bodies but not make any mechanistic conclusions for the reasons detailed below.

A) The critical experiment arguing that stress granules affect viral fitness in a manner influenced by NM120 is that the fitness difference in TRS+ (which makes NM120) and TRS- (which does not) is that the difference between these two viruses is less in a g3bp1Δ cell line when compared to WT cells (Figure 6J and K). However, I am concerned about this experiment since:

*i) The difference in fitness between recombinant viruses in g3bp1Δ cell line is observed only at 72hpi, which raises the concern that the result is not robustly different. What is the model for N*210 playing a role only at late infection time points?*

In a low MOI virus infection, several rounds of viral amplification are often required for maximal viral replication in a population of cells. Therefore, it can take one to several days for changes associated with a given viral replication cycle to become evident. After a low MOI infection (0.01), it was reported that SARS2 typically takes as little as 8 to 24 hours to complete its full replication cycle (Mautner & Hoyos, et al. 2022, Grebennikov, et al. 2021), a result that

illustrates the significant heterogeneity of SARS2 low MOI infections. 72 hours after a low MOI infection, SARS2 has likely completed multiple rounds of infections. We designed our co-infection competition experiments to proceed as multi-step growth experiments where infection of the monolayer is initiated by infection of few cells for two reasons. First, it minimizes the likelihood that two different viral mutants will co-infect the same cell (N^{*M210} produced by KR^{+TRS} may be able to *trans*-complement low N^{*M210}-producing viruses and confound our results). Second, a multi-step viral growth provides sufficient time to allow any replication advantage to reveal itself. Put another way, we do not think that the fitness difference that we see at 72hpi means that N^{*M210} enhances fitness at late infection times, and indeed a low MOI experimental setup does not permit us to make this conclusion because it is not a synchronous infection. Rather, the fitness difference provided by N^{*M210} is revealed by 72hpi because this is an asynchronous infection. As discussed in greater detail in later responses, we speculate that N^{*M210} may aid SARS-CoV-2 to establish an infection at early time points because it is a structural protein that is delivered to the cytoplasm of newly infected cells providing it with the opportunity to block G3BP1 foci formation at early infection times when other functionally redundant viral proteins are not yet present in significant amounts.

ii) A g3bp1Δ cell line will typically express G3BP2 and therefore still make stress granules, which would limit the conclusion that this effect is due to stress granules per se.

We would like to thank Dr. Parker for this extremely valid point. Taking this suggestion seriously, we performed new experiments to address this concern. The resubmission now contains new co-infection competition assays that were performed in ΔG3BP1/2 cells. Although ΔG3BP1 cells have reduced SG formation, ΔG3BP1/2 are devoid of canonical SGs, making them a superior choice to ask about the role of SGs in the fitness advantage of KR^{+TRS} (makes more N^{*M210}). KR^{+TRS} did not display a fitness advantage over KR^{-TRS} in ΔG3BP1/2 cells, while the fitness advantage was present in the matched control cells (new Fig 7I-J). These data add new mechanistic insight to our study, supporting our model that the fitness advantage of KR^{+TRS} is, in part, due to the ability of N^{*M210} to antagonize the formation of SGs and/or other G3BP1 foci. We thank Dr. Parker for this comment, as this new experiment has made our manuscript much stronger.

iii) In principle, the differences between a WT and g3bp1Δ cell line can be due to differences in G3BP1 function as opposed to stress granules per se.

Even considering the new co-infection competition experiments performed in ΔG3BP1/2 cells (Fig 7I-J), we agree with Dr. Parker. At present, our data cannot uncouple whether N^{*M210} provides a fitness advantage because it antagonizes the G3BP1/2 protein(s) directly or blocks G3BP1 foci. We have added text to the manuscript to acknowledge this limitation of our study (Lines 445-449, 670-674). However, as we discuss in the manuscript, it would be surprising for

SARS2 to produce a protein capable of blocking G3BP1 foci (Fig 6A-G, I-J), that does not co-immunoprecipitate with G3BP1 (Fig 6H) for the purposes of directly antagonizing a function of G3BP1 that is not related to the formation these foci. As Dr. Parker will appreciate, it is an ongoing challenge for any lab studying ribonucleoprotein granules to uncouple the function of a granule-associated protein from the function of its resident granule.

iv) We often observe differences in CRISPR edited cell lines that are not due to the targeted deletion and therefore using a rescue construct is important to conclude that the differences are due to the g3bp1Δ per se.

We agree with Dr. Parker, the possibility of off target or compensatory mutations is a significant limitation of any CRISPR knockout cell line that should be considered. In our studies, we have used different cell types (A549, primary HUVECs, and HEK293As) for co-infection competition assays, and KR^{+TRS} outcompeted KR^{-TRS} in all cell types. When testing knockout cell models, we obtained two different knockout cells: ΔG3BP1, produced by the Khaperskyy lab in a HEK293A background and ΔG3BP1/2 knockout cells produced by the Parker lab (shared by the Burke lab) in an A549 background. Although this does not directly address the concern of compensatory mutations, the consistent phenotypes observed in multiple cell lines and different knockout cells produced by different labs does provide some assurances that off target effects are not occurring. Unfortunately, because our Containment Level 3 facility has been closed since January 2025 for expansion and renovation, we were limited in the number of CL3 experiments that we could perform for this revision and as such, we were unable to complement the knockout cell lines. We have softened our conclusive statements and added a text about this limitation to the discussion (Lines 710-713).

B) I do think it is not great to consider any G3BP1 puncta as stress granules when it is now clear that in cells activating RNase L the assemblies are fundamentally different. If the authors want to continue to mix stress granules and RLBs, they should simply collectively refer to them as G3BP1 puncta.

We thank Dr. Parker for this comment and apologize for our inaccurate oversimplification of the RNA granule field in our original manuscript. In the revised manuscript, we have altered the text, figures, and graphical abstract to clearly define and differentiate RLBs (induced by poly I:C/RNase L) from SGs (induced by sodium arsenite [SA]). When SGs and RLBs are discussed together or cannot be definitively distinguished, we refer to them as G3BP1 foci.

2) It is notable that the authors observe that Nfl and NM120 inhibits stress granule formation with arsenite in a manner dependent on the dsRNA binding regions and by implication independent of the G3BP interacting domain. It would be important to note this is different from what has been previously described and provide a possible explanation.

We thank Dr. Parker for appreciating and highlighting what, to us, was one of the most surprising findings in our manuscript. We observe that N^{FL} and N^{*M210} block SA-induced SGs using a mechanism that relies on the dsRNA-binding motif (dsRBM) (now Fig 6I- J). Prior studies have reported that N^{FL} blocks SA-induced SGs, using a mechanism that requires the G3BP1-interacting domain (Luo, et al. 2021, Liu & Bai, et al. 2022, Biswal, et al. 2022). Yet in our study, $N^{FL\text{-}\Delta dsRBM}$ does not significantly decrease SGs, while N^{*M210}, which lacks the G3BP1-binding motif but retains the dsRBM, does. We do not suggest that the G3BP1 interaction is irrelevant; however, in our system we observe that the dsRBM is essential for N^{FL}-mediated SG block. Related observations have been made by other groups. Luo et al. (2021) observed that the G3BP1-N interaction is increased by RNA. It is possible that N^{FL} blocks SG formation using both mechanisms – binding to G3BP1 and blocking RNA-RNA bridging using the dsRBM. By contrast, N^{*M210} blocks SGs in the absence of any interaction with G3BP1 using a mechanism that requires the dsRBM (Fig 6H-J). In this case, we propose that N^{*M210} antagonizes RNA-RNA interactions that are required for SG formation (Parker et al, 2025), a theory that is consistent with the promiscuous ability of N^{*M210} to disassemble or block the formation of other ribonucleoprotein granules, including p-bodies and RLBs (Fig S5A-B, 6A-E). This is now discussed in the manuscript (Lines 607-612).

3) On many figures (especially quantifications), it would be helpful to have a title with distinguishing experiment characteristics (cell line, specific KO, etc. e.i. 6J and 6K, distinguishing WT and KO cell experiments). The figure panels should also be labeled to make it easy to interpret the figures.

We apologize for this oversight and have updated figures to increase understanding for the reader by including cell type and labeling the zoom panels more accurately. The following figures have been updated: Fig 3C, E, Fig 4D, Fig 6A, C, D, I, Fig 7, Fig S3B, Fig S4A, Fig S5A.

4) Quantifications of blots in figures 1, 2 to measure proportions of N isoforms would be helpful.

We thank Dr. Parker for this suggestion. We have added quantification of N* proteoforms to Figs 1, 2, S1 and S2.

5) Many of the IF images have Dapi signal scattered throughout the cytoplasm (see 6A for example). Is this a consequence of transient transfection? I worry about contamination.

We thank Dr. Parker for raising this important point. The extranuclear Hoechst -positive puncta are a result of transient DNA transfection. We have added a control experiment to the manuscript (Fig S3A) to illustrate that untreated cells, or those that have been mock transfected (no DNA)

lack the extranuclear Hoechst staining, while DNA-transfected cells do have cytoplasmic DNA puncta. We have added a line to the results (Lines 253-254) to explain this control.

Reviewer 2 Comments

The manuscript by Mulloy et al. is built on a previous observation, also reported by others, that certain SARS-CoV-2 variants —including Alpha, Gamma, and Omicron— have acquired two mutations within the N coding sequence (R203K/G204R), resulting in a novel canonical core sequence (CS) (ACGAAC). At the RNA level, this novel CS leads to the synthesis of a new subgenomic RNA (sgRNA-N), which encodes a truncated ORF named N*M210. At the protein level, prior studies have shown that these amino acid substitutions may affect the function of the N protein during infection, particularly its RNA binding activity.*

*A recent study by Mears et al. (PLoS Biol. 2025) demonstrated that the truncated N*M210 contributes to viral fitness during infection by antagonizing type I interferon production. This paper also described distinct phenotypes when the N coding sequence was mutated, compared to silent mutations that only ablate the TRS.*

*Mulloy et al. confirm and extend the findings of Mears et al. by investigating the mechanisms underlying the enhanced fitness of viruses expressing higher levels of the truncated protein N*M210. They hypothesised that N*M210 might sequester dsRNA in the cytoplasm, thereby preventing recognition by host pattern recognition receptors involved in IFN responses. This hypothesis is explored primarily through overexpression experiments assessing the impact on cellular pathways induced by dsRNA.*

*The manuscript provides novel insights into the mechanisms driving the fitness advantage of KR+TRS viruses that overproduce N*M210. First, they extensively demonstrate by in vitro experiments that N*M210 has a greater capacity to bind dsRNA than the full-length N protein. Furthermore, the superior fitness of KR+TRS viruses persists even in the absence of IFN responses, suggesting additional mechanisms beyond IFNß suppression. Then, they explore the formation of SGs, which have been associated with antiviral responses, using overexpression experiments.*

*1. While the study advances our understanding of the mechanisms of enhanced virus fitness when N*M210 is expressed during infection, many conclusions are based on overexpression experiments. To strengthen the physiological relevance, further validation using viral infection models is necessary.*

We thank the reviewer for their compliments and their recognition of the novelty and rigor of our study, particularly our overexpression analysis of N^{*M210}. We also agree with the reviewer that

to comprehensively understand the biology of a virus, researchers must use infectious virus, even if this requires extensive high-containment experimentation. There are two common methods to characterize the interplay between a viral protein and host cell biology: i) a reductionist approach using viral protein overexpression to elucidate the mechanism of action and ii) the production of recombinant viruses that eliminate or alter the viral protein of interest. Despite the essential nature of the N gene, a feature that makes it largely refractory to reverse genetics, we produced viral recombinants that manipulated the production of N^{*M210} to show that increased abundance of this protein provides SARS2 with a fitness advantage (several cell lines used, plus K18 mice, Figs 2F-M, S2J-L). Thus, overall, we have done both.

Although we used ectopic expression of N^{*M210} and N^{FL} to identify unique functions of N^{*M210}, most of these findings are well supported by both overexpression and infection experiments. Our dual approach of complementary overexpression and infection experiments is a consistent feature of the manuscript, for example: i) SARS2 variants produce truncated N* proteoforms in greater abundance (overexpression Fig 1K, S1E-F | infection Figs 1D-J, S1A-D, S1G), ii) N^{*M210} blocks IFN production and PKR activation (overexpression Fig5A-J | infection Fig 5L-M), iii) N^{*M210} induces p-body disassembly (overexpression Fig S5A-B | infection in our previously published manuscript Kleer, Mulloy *et al.* 2022. PLoS Path., Figs 1A-H, 2A-B).

However, Reviewer 2 makes a strong point regarding the imbalance of data that supports N^{*M210} blockade of G3BP1 foci. We acknowledge that the conclusions we made in the first submission of our manuscript about N^{*M210} preventing G3BP1 foci formation were derived largely from overexpression studies. In this resubmission, we have worked hard to rectify this imbalance and have added several new pieces of infection data to the revised manuscript.

i) We now provide evidence to show that G3BP1 foci form at early infection times with both KR^{+TRS} and KR^{-TRS} viruses. However, in cells where N^{FL} is limiting, KR^{+TRS} is superior at blocking G3BP1 foci formation compared to KR^{-TRS} (Fig 7B-E). It is not surprising that the impact of N^{*M210} is highest when N^{FL} is limiting. Although N^{*M210} is superior to N^{FL} at G3BP1 foci inhibition during overexpression (Fig 6A-G, I-J), N^{FL} is expressed ~5 times higher than N^{*M210} during infection (Fig 2D). Furthermore, because N^{*M210} can be packaged (Fig 1J), it is possible that N^{*M210} is particularly essential to diminish SGs/G3BP1 foci formation immediately after viral entry, functioning in a manner analogous to herpesviral tegument proteins to aid SARS2 in establishing an infection. These data support our model that N^{*M210} confers a fitness advantage, at least in part, by antagonizing G3BP1 foci, and suggest that N^{*M210} function may be particularly important at early infection times. These data are further discussed in the results (Lines 406-425) and discussion (Lines 624-638).

ii) As discussed, in response to Reviewer 1, we have added additional co-infection competition assays to the revised manuscript. In the original manuscript, we observed that in ΔG3BP1 cells,

there was a diminished fitness difference between KR^{+TRS} and KR^{-TRS} (now Fig 7F-H). To determine if this observation was reproducible or enhanced in SGs-null cells, we repeated competition assay experiments in ΔG3BP1/2 cells. We found that in these double knockout cells, the fitness difference between KR^{+TRS} and KR^{-TRS} was completely lost (Fig 7I-J). These infection data support the model that N^{*M210} antagonism of G3BP1 foci or the G3BP1 protein function is, in part, responsible for the observed fitness difference.

iii) We agree with the reviewer that it is important to consider physiological relevance. In our original manuscript, we conducted experiments in multiple systems: lung cell lines, primary human cells, and *in vivo* (K18 mice). However, to increase the relevance of our findings beyond model systems, we have added a new figure to the revised manuscript (Fig 8) wherein we analyzed SARS2 sequences from the human population over the last five years. Specifically, we tracked the origin and prevalence of viruses containing the novel N* TRS core sequence in the human population. We show that N* TRS-containing viruses have outcompeted non- N* TRS-containing viruses more than once. These data provide a real-world support of our reductionist studies and are discussed in the results (Lines 461-501) and discussion (Lines 685-699).

2. The discussion should clearly distinguish between results obtained from overexpression versus infection experiments, as this distinction is critical for interpreting the findings and assess their physiological relevance.

We thank the reviewer for this suggestion. We have added additional text to the manuscript to clearly distinguish data from infection versus overexpression experiments. For example, Lines 402 ("Overexpression of N^{*M210} potently inhibited G3BP1 foci, so we wondered if this remained true in the context of infection"), 511-512, 524, and 604.

*3. Line 331. "Despite the superior ability of N*M210 to bind and co-localize with dsRNA relative to NFL, in these assays we did not observe a significant difference in the ability of these two constructs to block dsRNA-induced responses". It is intriguing that overexpression assays (Fig. 5) show no significant differences in innate immune responses depending on dsRNA binding, such as phosphorylation of PKR and IRF3 and activation of RNaseL, between N*M210 and NFL, despite their differing dsRNA-binding capacities. Could the authors elaborate on this discrepancy?*

We thank the reviewer for this insightful comment, as we too were intrigued by this result. We believe there are two possible explanations for these data. i) In overexpression, we may have reached the saturation point of the assay, meaning that the expression level of N^{FL} and N^{*M210} is so high that, although N^{*M210} has higher dsRNA binding affinity, we are beyond the dynamic range to be able to resolve this difference in these assays. This possibility was discussed in the submitted version on the manuscript (Lines 552-554, in the discussion). ii) We now consider an

additional explanation for our data. It is possible that dsRNA binding affinity is not the limiting factor for optimal blockage of dsRNA responses. In other words, it is possible that N^{FL} has adequate dsRNA binding to inhibit poly I:C-induced antiviral responses such as PKR phosphorylation and activation of RNaseL. This second possibility was added to the discussion (Lines 554-556). "It is also possible that the weaker dsRNA binding ability of N^{FL} is nonetheless sufficient to inhibit poly I:C-induced antiviral responses."

*4. During authentic viral infection, increase din IFNβ production was observed when N*M210 was expressed (Fig. 5N), consistent with previous findings.*

We thank the reviewer for this comment. Although Mears et al 2025 showed that N^{*M210} overexpression diminished *IFNβ* production induced by poly I:C (Mears et al, Fig 6F), their manuscript did not examine *IFNβ* production during infection. To our knowledge, we are the first to show that viruses that make more N^{*M210} during infection are superior at blocking *IFNβ* production than viruses that make less. These data represent a significant advance, given the redundancy of interferon blocking viral proteins encoded by SARS2 (nsp1, nsp6, nsp7/8, nsp15, M, N^{FL}, orf3b, orf6, orf8, orf9b) (Reviewed in Setaro & Gaglia, 2021, and Sievers et al. 2024). Despite the wealth of viral proteins that diminish *IFNβ* that remain unchanged in the SARS2 recombinants, we showed that infection with viruses that make more N^{*M210} (KR^{+TRS}) produce less *IFNβ* that viruses that make less N^{*M210} (KR^{-TRS}). We were impressed to see that manipulation of N^{*M210} production during authentic infection influenced interferon response, enforcing the biological significance of this discovery.

...However, no significant differences were observed in PKR phosphorylation (Fig. 5M), suggesting that downstream processes dependent on PKR phosphorylation, such as SG formation, may also be unaffected. The proposed mechanism of inhibition of SG formation should be confirmed in infected cells.

We thank Reviewer 2 for this thoughtful comment. However, we would like to challenge the reviewer's statement that SG formation is likely to be dependent on PKR phosphorylation and activation. G3BP1 foci /SGs can be induced by both PKR-dependent and independent mechanisms. For example, SGs form independent of PKR activation after eIF4A inhibition, oxidative stress and activation of the unfolded protein response (UPR) via PERK (Reviewed by Baymiller & Moon, 2023). Burke et al. (2021) also showed that RLBs form in response to dsRNA but their formation does not require PKR. As SARS2 infection induces the UPR (Renner, et al. 2025, Caddell, et al. 2025 *pre-print*,), RLBs (Burke, et al. 2021, Fig S2E), and oxidative stress/elevated ROS (Cevallos, et al. 2025), there is significant literature to support that PKR activation and SG formation are not always coupled during SARS2 infection.

Our data reinforce the assertion that PKR activation status and G3BP1 foci formation are not always correlated. We confirm published literature (Long, et al. 2024) to show that during SARS2 infection, G3BP1 foci are detected only at early infection times and show that viruses with increased N^{*M210} production decrease G3BP1/TIAR foci in cells expressing low levels of N (Fig 7B-E).

5. Line 336. The statement "Both viruses induced an upregulation in total PKR and pPKR levels; however, pPKR levels were higher in cells infected with KR-TRS (Fig 5L & M)" should be revised. According to Fig. 5M, infection with the KR+TRS virus does not increase PKR phosphorylation compared to mock-infected cells, whereas a significant increase was observed when the TRS was ablated (KR-TRS).

We thank Reviewer 2 for catching this mistake. We have revised the text interpretation of Fig 5L-M to more accurately reflect the data. In these experiments, only infection with KR^{-TRS}, and not KR^{+TRS}, statistically increased p-PKR/PKR compared to mock infected cells.
Revised text now reads:
"Infection with both KR^{+TRS} and KR^{-TRS} induced PKR activation (pPKR/PKR) as evidenced by increased abundance of phosphorylated PKR after immunoblotting (Fig 5L). However, only infection with KR^{-TRS}, not infection with KR^{+TRS}, significantly increased phospho-PKR levels relative to mock conditions (Fig 5M), suggesting KR^{+TRS} displays a superior ability to suppress PKR activation during infection." Lines 331-334.

6. Some of the antiviral responses induced by dsRNA, such as PKR and RNaseL activation, lead to translation inhibition. It would be valuable to assess whether translation is differentially affected during KR+TRS vs KR-TRS infection.

We thank the reviewer for this interesting comment. To test this, we used a puromycilation assay to measure active protein synthesis during infection (see below). Briefly, we fed puromycin to cells infected with either KR^{+TRS} or KR^{-TRS}. When puromycin blocks protein translation, it is incorporated into nascent polypeptides, 'tagging' arrested translation products, and permitting their visualization with an anti-puromycin antibody and standard western blotting approaches. In our preliminary experiment, shown below, we did not observe any significant difference in nascent polypeptide production between cells infected with KR^{+TRS} versus KR^{-TRS}. However, in this preliminary experiment, we have not captured the expected SARS2-mediated translation block, either because the MOI was too low or the infection time was too early. Thus, although these preliminary data suggest that enhanced production of N^{*M210} is insufficient to influence bulk protein synthesis, we cannot yet make any strong conclusions from these data. We are currently conducting a separate follow-up study to characterize if N^{*M210} influences the translation of selected mRNAs. However, we view this as a distinct research question and beyond the scope of this manuscript.

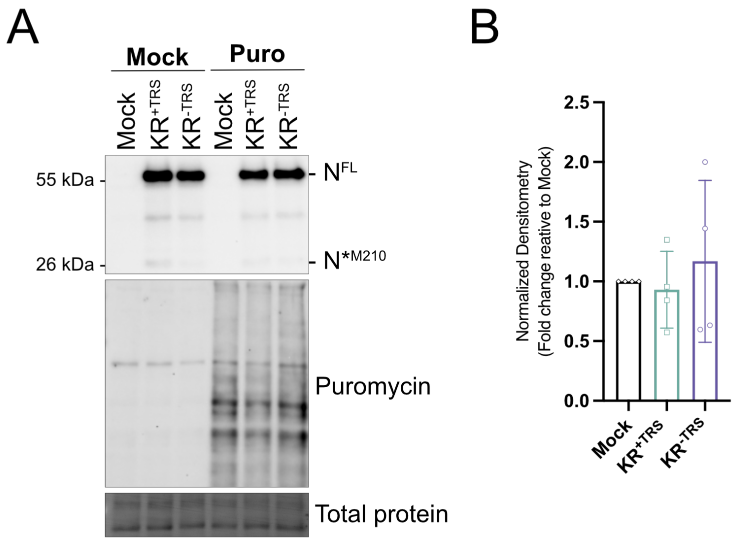

KR^{+TRS} does not differentially alter bulk translation compared to KR^{-TRS}. A. $A549^{ACE2-TMPRSS2}$ cells were infected with rSARS-CoV-2 KR^{+TRS} or KR^{-TRS} (MOI=2), or mock infected. 24 hpi, cells were treated with 10 µg/mL puromycin at 37˚C for 5 min, before cells were lysed. Proteins were resolved by immunoblotting using an anti-N antibody and an anti-puromycin antibody. Puromycin densitometry was normalized to total protein and graphed relative to mock-infected cells. n=4.

*7. Line 384. Based on overexpression experiments, the authors conclude that "Given that N*M210 blocks dsRNA-induced SGs (Fig 6) and inhibits PKR activation (Fig 5), and both phenomena required the dsRBM, we predicted that N*M210 achieves both via sequestering the SG inducer, dsRNA, preventing PKR activation and subsequent SG formation". However, both N*M210 and N-FL inhibit PKR phosphorylation to a similar extent (Fig. 5C), while only N*M210 inhibits polyI:C-induced SG formation. This point warrants further discussion.*

We thank Reviewer 2 for this comment, and have addressed it, in part, by other requested revisions to the manuscript.

i) In response to Reviewer 1's comments, we have now revised the manuscript to differentiate between RLBs, which form in response to dsRNA, and SGs, which form after sodium arsenite stress. In the experiments shown in Fig 6A-G), the G3BP1 foci blocked by N^{*M210} are likely RLB, not SGs. RLB formation does not require PKR activation but does require RNase L activation (Burke et al., 2021).

ii) As discussed, PKR activation status and RLB formation are not always correlated. Even if the phenomena of PKR inhibition and RLB blockade were both occurring because N^{*M210} acts as a sponge, binding the dsRNA inducer, it does not follow that they must both require the same stoichiometry for an effect. In other words, the threshold level of dsRNA needed to activate PKR

is likely to be different than that required to induce RLB formation via RNaseL activation. Similarly, the threshold level of N^{*M210} required to block these two phenotypes may likewise differ.

That said, we have carefully considered the reviewer's point in the context of the literature which says that RLB formation does not require PKR but does require RNaseL (Burke et al. 2021). N^{FL} and N^{*M210} block RNase L activation to a similar extent (Fig 5I); therefore, why is N^{*M210} so much better at blocking RLBs when RLBs require RNaseL activation? We propose that N^{*M210} can antagonize RLBs in two distinct ways: first, like N^{FL}, N^{*M210} binds the inducer, dsRNA, to block RNase activation and second, N^{*M210} prevents RNA-RNA interactions that are necessary for most ribonucleoprotein granules to form (Parker, et al. 2025). This is consistent with our observations that N^{*M210} blocks other RNA granules (P-bodies, Fig S5A-B) and other G3BP1 foci (SGs induced by arsenite stress, Fig 6I-J), independent of their mode of induction, but dependent on the N^{*M210} dsRBM. By contrast, only one of the above mechanisms, N^{*M210} binds the inducer dsRNA, will block activation of RNase L. We have added additional text to the discussion to reflect this theory (Lines 616-623).

*8. While overexpression experiments show that N*M210 inhibits polyI:C-induced SG formation, its relevance during infection remains unconfirmed. Co-infection competition assays in G3BP1 KO cells, which are impaired for SG formation, suggest forshowed a decrease in the fitness advantage of KR+TRS virus, suggesting that SG inhibition may contribute to viral fitness. However, additional infection-based evidence is needed.*

We thank the reviewer for this suggestion. In response to comments from both reviewers, we have provided new experimental data performed during infection to support and enhance our conclusions. The following additional infection experiments have been added to the manuscript.

i) We conducted addition co-infection competition assays in ΔG3BP1/2 cells that lack the ability to produce canonical SGs. These data show that the fitness advantage of KR^{+TRS} (makes more N^{*M210}) over KR^{-TRS} (makes less N^{*M210}) is lost that ΔG3BP1/2 cells (Fig 7I-J), strengthening our conclusion that N^{*M210} promotes viral fitness in part by antagonizing the formation of G3BP1 foci.

ii) We now show that G3BP1 foci form during SARS-CoV-2 infection at early time points. Capitalizing on our ability to find G3BP1 foci during infection, we show that in low N expressing cells, viruses that produce more N^{*M210} (KR^{+TRS}) are superior to viruses that produce less (KR^{-TRS}) at blocking the formation of G3BP1 foci (Fig 7B-E). This experiment shows that even though N^{FL} is produced in much greater abundance than N^{*M210} during infection, N^{*M210} aids in G3BP1 foci blockade at early infection times, a time when the virus is most at risk of defeat by cellular antiviral responses.

iii) We conducted an additional co-infection competition assay between WT and KR^{-TRS} viruses (Fig 2L). Both viruses make only low levels of N^{*M210}, but KR^{-TRS} harbours the amino acid change in N^{FL} that has become fixed in the population. These data show that there is no significant fitness advantage provided by the KR amino acid substitution, reinforcing our conclusion that the fitness advantage in KR^{+TRS} is due to its ability to produce the N*sgRNA and N^{*M210} in greater abundance.

iv) We conducted an analysis of the prevalence of the novel N* TRS core sequence in the human population over time. These real-world data show viral variants containing the N* TRS emerged, became fixed in the population, and were then transiently lost in late 2024, only to re-emerge and dominate again in 2025 (Fig 8B-D).

Taken together, the abundant additional infection data (Figs 2L, S5C-D, 7A-E, 7I-J, 8A-D, S6A-B) added to the manuscript have strengthened our manuscript and we thank the reviewer for the suggestion.

Minor points

1. Line 84. "sgRNA synthesis is governed by the presence of short ~6 nucleotide sequences, called transcription regulatory sequences (TRSs)". This sentence and the corresponding figure (Fig. 1) should be revised. The 6-nt conserved sequence is the core sequence (CS), while TRSs include the CS and 5' and 3' flanking sequences, which also regulate transcription.

We apologize for this mistake and thank the reviewer for bringing it to our attention. The text (Lines 77, 466, 470, 491) and figure captions (Figure 1 2, & 8) have been modified to clarify that we are discussing the TRS core sequence/hexamer.

2. While many viruses inhibit SGs and P-bodies during infection, some transiently induce SGs or promote their formation to enhance replication, such as RSV (Linquist et al., 10.1128/JVI.00260-10). This should be acknowledged.

We thank the reviewer for this comment. We have added a statement to the manuscript to indicate that not all viruses block the formation of G3BP1 foci (Lines 579-581).

3. Line 564. The discussion highlights the reverse genetics approach used to dissect transcriptional versus protein-level effects. However, Mears et al. (PLoS Biology 2025) previously addressed this using a similar strategy and should be cited.

We thank Reviewer 2 for this thoughtful comment. We did cite Mears et al. 2025 in our submitted manuscript; however, we have added additional citations and discussion of this study in the revised manuscript. This includes text acknowledging that Mears et al. used a similar strategy to our own to build recombinant SARS2 viruses (Lines 675-676).

Though we believe it is ethical and important to acknowledge the work of others, especially in this case, it may assuage the reviewer's concerns to know that our study was largely completed before the end of 2024, our manuscript was posted as a preprint in Feb 2025, a few weeks after Mears et al. was first published, and our paper was submitted to PLoS Biol within the 6 months allocated for 'scoop protection' by this journal.
