# Supplementary material for: Evolution of a truncated nucleocapsid protein enhances SARS-CoV-2 fitness by suppressing antiviral responses
Source: PLoS Biol. 2026 Apr 1;24(4):e3003646. doi: 10.1371/journal.pbio.3003646 (PMC13043052; doi:10.1371/journal.pbio.3003646)
Supplement: Attachment — Submitted filename: Response to editors comments.pdf [file pbio.3003646.s020.pdf]

Response to Editor's Comments

1. We routinely suggest changes to titles to ensure maximum accessibility for a broad, non-specialist readership, and to ensure they reflect the contents of the paper. In this case, we would suggest a minor edit to the title, as follows. Please ensure you change both the manuscript file and the online submission system, as they need to match for final acceptance:

"Evolution of a truncated nucleocapsid protein enables SARS-CoV-2 to suppress dsRNA-triggered antiviral defenses"

We love the idea of adding the evolutionary angle to the title; however, we propose a slight modification to the title suggested by the editor. We prefer: "Evolution of a truncated nucleocapsid protein enhances SARS-CoV-2 fitness by suppressing antiviral responses". We think this title will improve reader accessibility while still highlighting our main discoveries with accuracy. There are two reasons why we would prefer not to put "dsRNA-triggered" in the title. First, in the manuscript our most significant finding is that N^{*M210} confers a fitness advantage, largely because of its ability to inhibit stress granules (SGs). Because N^{*M210} blocks SGs that form after the inducer sodium arsenite, not only after the inducer dsRNA, the mechanism utilized by N^{*M210} to block SGs cannot solely occur via binding of the dsRNA inducer. In other words, using its dsRNA-binding domain, N^{*M210} likely binds short dsRNA or structured RNA that are required for SGs to form. This model is also supported by the ability of N^{*M210} to disassemble unrelated RNA granules called p-bodies. Second, although N^{*M210} blocks all dsRNA-triggered antiviral responses tested (interferon production, RNaseL activation, PKR activation), its ability to do so was not significantly greater than N^{FL} in ectopic overexpression models. These data, along with competition assays in interferon-defective Vero cells, wherein viruses that make more N^{*M210} still outcompete those that make less, suggest that the fitness advantage provided by N^{*M210} does not rely on blockade of the dsRNA-triggered interferon response. Rather, our data suggests that blocking the formation of antiviral SGs (whether elicited by dsRNA or another inducer) is the most important function elicited by N^{*M210}.

2) Please add weblink to the funding agencies in the Financial Disclosure statement in the manuscript details.

The following information has been added to the manuscript acknowledgements section and to the Financial Disclosure Statement.

Operating funds were awarded from the Institute of Infection and Immunity of the Canadian Institutes for Health Research (https://cihr-irsc.gc.ca/e/13533.html) (#195645).

An operating grant (#175622) awarded to the Coronavirus Variants Rapid Response Network (CoVaRR-Net; https://covarrnet.ca/) from the Institute of Infection and Immunity of the

Canadian Institutes of Health Research (https://cihr-irsc.gc.ca/e/13533.html). JAC and LF were members of CoVaRR-Net.

The funders had no role in study design, data collection and analysis, decision to publish, or preparation of the manuscript.

3) The Ethics statement needs to be a separate, independent (and the first) subheading in the Material & Methods section. It must include the full name of the IACUC/ethics committee that reviewed and approved the animal care and use, as well as the protocol/permit/project license number.

An ethics statement subheading with all relevant information has been added to the Materials and Methods section (Lines 805-809) and reads as follows. "Animal work was conducted under CL3 conditions with authorization from the CRCHU de Quebec-Université Laval animal care committee (#2025-1709) were approved by the Comité de Protection des Animaux de l'Université Laval (CPAUL-3)."

3) … Please supply the numerical values either in the a supplementary file or as a permanent DOI'd deposition for the following figures:
Figure 1GI, 2DF-M, 3BDFG, 4E, 5BCDFGHMNO, 6BEFGJ, 7CE-J, 8BCD, S1A-D, S2BCE-M, S3C, S4B, S5BCD, S6AB
NOTE: the numerical data provided should include all replicates AND the way in which the plotted mean and errors were derived (it should not present only the mean/average values).

We have provided an excel spreadsheet that includes all raw numerical value source data for these figures and supplemental figures. We did not include the numerical values for Figure 8BCD and Figure S6AB in this file, as these graphs were generated using RStudio from large genome libraries acquired from NextStrain.org. These source data are not of our generation and are publicly available. Source code is available at github https://github.com/rmulloy97/Mulloy-et-al.-SARS-CoV-2-TRS-prevalence or Zenodo.org DOI 10.5281/zenodo.18225476.

4) Please cite the location of the data clearly in all relevant main and supplementary Figure legends…

We have added details regarding where the source data/extended data can be found to the figure captions of all relevant figures.

5) We require the original, uncropped and minimally adjusted images supporting all blot and gel results reported in the Figures 1DEFHJK, 2BC, 3A, 4AF, 5AEIJL, 6H, S1EFG, S2AD, S5E

Original, uncropped blots can be found in the pdf file, Extended_data_S1_raw_images, uploaded with supporting information.

6) We will require these files before a manuscript can be accepted so please prepare and upload them now. Please carefully read our guidelines for how to prepare and upload this data: https://can01.safelinks.protection.outlook.com/?url=https%3A%2F%2Fjournals.plos.org%2Fplosbiology%2Fs%2Ffigures%23loc-blot-and-gel-reporting-requirements&data=05%7C02%7Cjennifer.corcoran%40ucalgary.ca%7Caa4503f9a139485fd43b08de4e2f8615%7Cc609a0eca5e346319686192280bd9151%7C1%7C0%7C639034163204550288%7CUnknown%7CTWFpbGZsb3d8eyJFbXB0eU1hcGkiOnRydWUsIlYiOiIwLjAuMDAwMCIsIlAiOiJXaW4zMiIsIkFOIjoiTWFpbCIsIldUIjoyfQ%3D%3D%7C0%7C%7C%7C&sdata=UZpL2W58P41ffObQcfey1b0O0llJ9ZrzniZYwLvDhIs%3D&reserved=0

7) Please add a scale bar in all microscopy pictures

All microscopy figures contain a scale bar. All image panels within a given figure were subject to identical imaging and cropping conditions without use of digital zoom; therefore, the scale bar provided on the control panel applies to all panels.

8) Please ensure that your Data Statement in the submission system accurately describes where your data can be found and is in final format, as it will be published as written there.

A data availability subheading was added to the methods section of the manuscript (Lines 1210-1211). "Recombinant SARS-CoV-2 sequences can be accessed on NCBI: accession numbers: WT: PV935594.1, KR^{+TRS}: PV952734, KR^{-TRS}: PV936453, M210I: PV955084. Rscript pipeline for SARS-CoV-2 mutational analysis (Figure 8 & S6) can be accessed on github https://github.com/rmulloy97/Mulloy-et-al.-SARS-CoV-2-TRS-prevalence or Zenodo.org DOI 10.5281/zenodo.18225476. All other data underlying the findings in this study are available in supporting information (Extended Data or Source Data files)."

9) Please note that we cannot accept sole deposition of code in GitHub, as this could be changed after publication. However, you can archive this version of your publicly available GitHub code to Zenodo.

We have uploaded all code into Zenodo (Zenodo.org DOI 10.5281/zenodo.18225476) and added this to the methods section.
